# Evaluating Probe Design for Phylogenomics Across Taxonomic Scales: First Steps for Applying Ultraconserved Elements in an Understudied Class (Mollusca: Polyplacophora)

**DOI:** 10.1111/1755-0998.70076

**Published:** 2025-11-13

**Authors:** Zeyuan Chen, Katarzyna Vončina, Julia D. Sigwart

**Affiliations:** ^1^ Department of Marine Zoology Senckenberg Research Institute and Natural History Museum Frankfurt Frankfurt Germany; ^2^ Department of Biological Sciences, Institute of Ecology, Evolution and Diversity Johann Wolfgang Goethe University Frankfurt Frankfurt Germany

**Keywords:** marine biodiversity, mollusca, museomics, phylogenomics, polyplacophora, ultraconserved elements

## Abstract

Ultraconserved elements (UCEs) have become a powerful tool for phylogenomics, but probe sets optimized for one lineage often perform inconsistently when applied in others. Here, we designed and tested new UCE probe sets derived from both genome and transcriptome data of an understudied molluscan class, Polyplacophora (chitons). In this study, we identified 5730 ultra‐conserved elements (UCEs) from available chiton genomes and transcriptomes, and designed a set of 19,080 probes. These probes showed an average efficiency of 55% in the genome and 20% in transcriptomes, significantly outperforming available molluscan probe sets. A coalescence‐based phylogenetic tree based on *in silico* extractions of UCEs from transcriptome and genome data successfully resolved chiton phylogeny at the species level. Relatively shorter flanking regions performed best. Where genome and transcriptome data were available for the same species, they did not always resolve as sister taxa in non‐optimized methods; instead, genome‐ and transcriptome‐derived sequences tended to form separate clades. This offers a caution for combining data harvested from published datasets. Quantifying phylogenetic signal at individual UCE loci demonstrates that the dataset retains topological stability across a range of filtering stringencies. This resource provides a foundation for integrating new genomic and transcriptomic datasets and has the potential to enable targeted sequencing of historical museum specimens. More broadly, our study highlights the importance of tailored probe design for phylogenomic studies in understudied lineages and the challenges of combining diverse molecular data types.

## 1 Introduction

The extremely low variability of ultra‐conserved elements (UCEs) allows these regions and the flanking sequences to be efficiently captured by probes across species, and the variability within the flanking sequences is phylogenetically informative (Faircloth et al. 2012; Gilbert et al. 2015; Edwards et al. 2017). By means of target enrichment sequencing, this new approach has successfully recovered the phylogeny of mammals (McCormack et al. 2012), fish (Faircloth 2017), birds (McCormack et al. 2016), insects (Baca et al. 2017; Branstetter et al. 2017; Starrett et al. 2017), corals (Quattrini et al. 2018), flowering plants (Zuntini et al. 2024), mosses (Liu et al. 2019) and others. Low requirements of DNA, batch operation and low costs make UCE approaches relatively easy to apply to a large number of species. For example, in angiosperms, this has been used to reconstruct a phylogeny sampling almost 8000 genera (Zuntini et al. 2024). UCEs are increasingly favored in large‐scale phylogenomic studies, showing strong potential to resolve phylogeny at multiple evolutionary scales (Faircloth et al. 2012), recover difficult nodes (Gilbert et al. 2015; Esselstyn et al. 2017; Quattrini et al. 2018; Stiller et al. 2024), and extract usable sequence data from preserved museum specimens (McCormack et al. 2016; Ruane and Austin 2017; Derkarabetian et al. 2019). Recently, UCE probes have also been successfully designed and applied to reconstruct phylogenies of heterobranch gastropods and bivalves, two of the most species‐rich groups within molluscs (Moles et al. 2021; González‐Delgado et al. 2024; Li et al. 2024). Previous work (e.g., Lemarcis et al. 2025) has demonstrated that probe sets can be transferred across lineages within gastropods, while also showing that capture efficiency decreases gradually with increasing genetic distance rather than at a distinct threshold. This raises the practical question of transferability across molluscan clades: given that UCEs are, by definition, highly conserved, can UCE probe sets designed for gastropods or bivalves be effectively used in another taxonomic class of molluscs, or is a new probe set required for effective phylogenetic reconstruction?

Mollusca is the second‐largest animal phylum with about 130,000 extant species (Haszprunar et al. 2008; Vinther 2015; Slater and Bohlin 2022). They are divided into two main clades: Conchifera, comprising most shelled molluscs together with cephalopods, and Aculifera, comprising the worm‐molluscs and chitons. Chitons (Polyplacophora) are of particular interest in evolutionary biology as they have a remarkable degree of morphological conservatism characterised by eight aragonitic shell valves (Sigwart 2017; Wanninger and Wollesen 2019). Despite apparent morphological stability over spans of hundreds of millions of years, there are over 1000 extant species of chitons. Living species exhibit an abundance of sensory adaptations (Moseley 1885; Sigwart et al. 2010; Chappell and Speiser 2023; Ampuero et al. 2024), and highly variable genome architecture with rapid and frequent chromosomal rearrangements (Sigwart et al. 2025).

Living chitons are divided into three orders: Lepidopleurida, Callochitonida, and Chitonida (Giribet and Edgecombe 2020). Lepidopleurida comprises primarily deep‐sea species with distinctive morphological synapomorphies, such as aesthete arrangement, gill structure, and the specialised Schwabe Organ, all of which show significant morphological and molecular divergence (Sigwart et al. 2014). Callochitonida is the sister lineage to Chitonida (Giribet and Edgecombe 2020; Irisarri et al. 2020; Moles et al. 2021), and together they are considered Chitonida *sensu lato*. Chitonida *sensu stricto*, which includes nearly 80% of extant chiton species, is further divided into two suborders: Chitonina (comprising the superfamilies Chitonoidea and Schizochitonoidea) and Acanthochitonina (comprising the superfamilies Mopalioidea and Cryptoplacoidea) (Sirenko 2006). Chiton taxonomy and systematics are based on a broad suite of features including shell structure, radula characters, girdle armature, egg and sperm characteristics. Many characters are confoundingly variable and/or based on microscopic features, which have hindered species discrimination and internal phylogenetic resolution. New species continue to be discovered and described regularly.

The prevailing molecular markers such as mitochondrial genes are unable to resolve the relationships below the rank of superfamily (Irisarri et al. 2020; Liu et al. 2023), the use of transcriptome and genome data shows a relatively stable topology for superfamily‐level groups; however, the positions of some families are still unstable (Liu et al. 2023). Moreover, the high requirements of transcriptome and genome data for the preservation status of the samples, DNA quality/quantity, high cost, long time cycles and large computing resources also limit the reconstruction of large‐scale chiton phylogeny with adequate taxon sampling for this large clade. Major questions remain about understanding the diversity within chitons, and the fine‐scale phylogeny within and among families. Meanwhile, the preservation of chitons as dry tissue in museum collections, including many rare species, has been noted as an invaluable resource for potential museomics and extraction of sequence data to increase taxon sampling in molecular phylogenetics (Vončina and Sigwart 2025).

There is an established backbone phylogeny for chitons (Liu et al. 2023), but less than 30% of living species have been sampled even for barcode sequences (Ratnasingham and Hebert 2007); a UCE probe set would be an important step forward to unlock the vast material available in museum collections. In this study, we found that although probes designed for bivalves and heterobranch gastropods can capture hundreds of UCE loci in chitons, these loci are insufficient to recover chiton internal relationships, highlighting the necessity of designing clade‐specific probe sets. In the present study, we designed chiton‐specific hybrid probes from available genomes and transcriptomes, and tested their ability to recover order to species‐level phylogenetic relationships within chitons. This presents an opportunity to test the effectiveness of novel UCE probe sets compared to available resources—generating important knowledge that is transferable to other understudied groups.

## 2 Materials and Methods

Previously published and novel genome and transcriptome data were used to create probes and were used *in silico* to compare the performance of different flanking regions and evaluated based on the reliability of the phylogenetic reconstruction produced.

### 2.1 Preparation of Source Genomes and Transcriptomes

All the available chiton genome assemblies and RNA‐seq reads were downloaded from NCBI (Table 1 and Table S1). In total, there are 18 chiton genomes and 28 transcriptomes, mostly representing unique species, with 10 examples where both genome and transcriptome data are available for the same species. Both genome and transcriptome datasets cover all three orders of chitons: Lepidopleurida, Callochitonida, and Chitonida.

**TABLE 1:** Genomes and transcriptomes used in probe design and *in silico* analyses.

Order	Family	Species	Location	Total length of assembly	No. of contigs	Contig N50	BUSCOs	Genome accession number	Assembly source	Biosample	# Loci recovered				
											GP: 9177 probes; 3271 loci	EP: 9903 probes; 2459 UCE	MP: 19020 probes; 5730 loci	HP: 57,606probes	BP: 19,657probes
*Genome*															
Callochitonida	Callochitonidae	*Callochiton septemvalvis*	Northern IrelanD: Kircubbin, Strangford Lough	1,229,767,851	3599	608,062	C: 94.4%[S: 91.9%, D: 2.5%], F: 2.6%, M: 3.0%, n: 954	GCA_047826735.1	(Sigwart et al. 2025)	SAMN41482309	1899	675	2360	1233	848
Chitonida	Acanthochitonidae	*Acanthochitona discrepans*	Northern IrelanD: Kircubbin, Strangford Lough	1,173,574,984	951	3,476,547	C: 97.5%[S: 95.0%, D: 2.5%], F: 1.3%, M: 1.2%, n: 954	GCA_048544545.1	(Sigwart et al. 2025)	SAMN41504957	2446	602	2800	1289	902
Chitonida	Acanthochitonidae	*Acanthochitona rubrolineata*	China: Qingdao	1,093,798,424	227	24,072,763	C: 97.5%[S: 95.7%, D: 1.8%], F: 1.8%, M: 0.7%, n: 954	GCA_045838495.1	(Qu et al. 2024)	SAMN41482308	2344	602	2715	1340	874
Chitonida	Chitonidae	*Acanthopleura granulata*	USA: Florida Keys, Tavernier	544,712,841	5936	1,098,986	C: 96.0%[S: 95.3%, D: 0.7%], F: 2.1%, M: 1.9%, n: 954	GCA_016165875.1	(Varney et al. 2021)	SAMN13050533	2178	634	2597	1336	1397
Chitonida	Mopaliidae	*Cryptochiton stelleri*	USA:Washington State, San Juan Island	782,962,633	365,105	3056	C: 13.7%[S: 13.3%, D: 0.4%], F: 47.8%, M: 38.5%, n: 954	GCA_031471745.1	—	SAMN34510629	2858	1024	3600	1062	776
Chitonida	Mopaliidae	*Katharina tunicata*	USA:Washington State, San Juan Island	681,755,286	244,286	5405	C: 54.9%[S: 54.6%, D: 0.3%], F: 33.2%, M: 11.9%, n: 954	GCA_032466195.1	—	SAMN34510619	2904	1174	3779	1143	841
Chitonida	Tonicellidae	*Lepidochitona cinerea*	United KingdoM: Cornwall, Mount Edgcombe, Barn Pool	740,361,436	204	8,912,738	C: 96.6%[S: 93.0%, D: 3.6%], F: 2.4%, M: 1.0%, n: 954	GCA_963971465.1	—	SAMEA12219462	2195	612	2597	1097	734
Chitonida	Ischnochitonidae	*Lepidozona retiporosa*	USA:Washington State, San Juan Island	502,681,105	302,112	2234	C: 30.8%[S: 28.9%, D: 1.9%], F: 32.2%, M: 37.0%, n: 954	GCA_032360285.1	—	SAMN35990794	438	22	428	249	45
Chitonida	Chitonidae	*Liolophura sinensis*	Hong Kong:Kau Sai Chau	609,273,693	1742	764,463	C: 96.4%[S: 95.8%, D: 0.6%], F: 1.8%, M: 1.8%, n: 954	GCA_032854445.2	(Hong Kong Biodiversity Genomics Consortium et al. 2024)	SAMN35152372	2239	667	2671	1353	1369
Chitonida	Mopaliidae	*Mopalia ciliata*	USA:Washington State, San Juan Island	661,751,784	206,667	5022	C: 27.3%[S: 27.1%, D: 0.2%], F: 53.9%, M: 18.8%, n: 954	GCA_034783835.1	—	SAMN34510622	2950	1086	3728	1167	844
Chitonida	Mopaliidae	*Mopalia kennerleyi*	USA:Washington State, San Juan Island	742,905,398	266,591	4396	C: 30.6%[S: 29.6%, D: 1.0%], F: 48.3%, M: 21.1%, n: 954	GCA_034783855.1	—	SAMN34510623	2872	1042	3618	1086	805
Chitonida	Mopaliidae	*Mopalia muscosa*	USA:Washington State, San Juan Island	661,527,017	169,779	6693	C: 32.7%[S: 32.5%, D: 0.2%], F: 50.4%, M: 16.9%, n: 954	GCA_031763545.1	—	SAMN33059285	2969	1084	3742	1161	839
Chitonida	Mopaliidae	*Mopalia swanii*	USA:Washington State, San Juan Island	858,093,624	537,259	2201	C: 20.9%[S: 19.5%, D: 1.4%], F: 53.0%, M: 26.1%, n: 954	GCA_030265315.1	—	SAMN33070946	2633	926	3287	888	664
Chitonida	Mopaliidae	*Mopalia vespertina*	USA:Washington State, San Juan Island	674,984,218	169,587	6583	C: 37.2%[S: 36.8%, D: 0.4%], F: 45.4%, M: 17.4%, n: 954	GCA_030265115.1	—	SAMN33070948	2963	1080	3740	1162	863
Chitonida	Tonicellidae	*Tonicella lineata*	USA:Washington State, San Juan Island	920,189,567	459,948	2841	C: 15.1%[S: 14.8%, D: 0.3%], F: 48.7%, M: 36.2%, n: 954	GCA_034781015.1		SAMN35990815	2872	1038	3621	1095	791
Chitonida	Tonicellidae	*Boreochiton ruber*	Northern IrelanD: Kircubbin, Strangford Lough	1,660,742,690	825	4,032,827	C: 96.6%[S: 94.7%, D: 1.9%], F: 2.7%, M: 0.7%, n: 954	GCA_048127235.1	(Chen et al. 2025)	SAMN41706136	2842	1089	3642	1177	831
Lepidopleurida	Protochitonidae	*Deshayesiella sirenkoi*	Western Pacific Ocean: Daikoku vent field	1,546,223,685	903	4,972,616	C: 97.1%[S: 96.4%, D: 0.7%], F: 1.9%, M: 1.0%, n: 954	GCA_045838535.1	(Sigwart et al. 2025)	SAMN41482322	1888	980	2656	1340	855
Lepidopleurida	Hanleyidae	*Hanleya hanleyi*	Norway: Bergen	2,516,596,252	57,704	64,677	C: 83.3%[S: 78.1%, D: 5.2%], F: 11.6%, M: 5.1%, n: 954	GCA_036873755.1	(Varney et al. 2022)	SAMN26529200	1701	868	2370	1141	735
Stylommatophora (Gastropoda)	Xanthonychidae	*Polymita picta*	Cuba	2,723,727,283	55,021	93,682	C: 88.4%[S: 81.4%, D: 7.0%], F: 7.3%, M: 4.3%, n: 954	PRJNA1250545	(Reyes‐Tur et al. 2025)	SAMN47943309	780	93	817	730	179
Stylommatophora (Gastropoda)	Arionidae	*Arion vulgaris*	Germany	1,540,993,941	7076	8,603,329	C: 94.5%[S: 88.3%, D: 6.2%], F: 2.6%, M: 2.9%, n: 954	GCA_020796225.1	(Chen et al. 2022)	SAMN16874494	401	72	440	1164	325
*Transcriptome*															
Callochitonida	Callochitonidae	*Callochiton septemvalvis*	Spain: Tossa de Mar	143,253,018	126,149	1920	C: 97.8%[S: 52.7%, D: 45.1%], F: 2.0%, M: 0.2%, n: 954	Ref Table S1		SAMN16677970	682	398	990	386	307
Callochitonida	Callochitonidae	*Callochiton* sp.	Antarctica: Ross Sea	35,702,143	58,279	926	C: 29.7%[S: 24.9%, D: 4.8%], F: 21.0%, M: 49.3%, n: 954	Ref Table S1		SAMN14765619	194	659	736	225	145
Chitonida	Acanthochitonidae	*Acanthochitona crinita*	France: Coast Roscoff	133,608,902	110,569	2330	C: 99.9%[S: 56.8%, D: 43.1%], F: 0.1%, M: 0.0%, n: 954	Ref Table S1		SAMN06141845	1069	482	1403	625	419
Chitonida	Acanthochitonidae	*Acanthochitona discrepans*	Northern IrelanD: Kircubbin, Strangford Lough	96,253,736	145,509	1131	C: 60.4%[S: 47.0%, D: 13.4%], F: 7.8%, M: 31.8%, n: 954	Ref Table S1		SAMN41504957	748	411	1008	464	297
Chitonida	Acanthochitonidae	*Acanthochitona rubrolineata*	China: Qingdao	316,833,138	274,164	2335	C: 100.0%[S: 25.5%, D: 74.5%], F: 0.0%, M: 0.0%, n: 954	Ref Table S1		SAMN10743605‐06	603	184	741	197	142
Chitonida	Chitonidae	*Acanthopleura gemmata*	Australia: Queensland, Heron Island	156,499,571	249,299	970	C: 88.0%[S: 50.4%, D: 37.6%], F: 7.4%, M: 4.6%, n: 954	Ref Table S1		SAMN14765616‐17	654	414	947	454	471
Chitonida	Chitonidae	*Acanthopleura granulata*	USA:Florida, Florida Keys, near Harry Harris State Park	149,570,244	198,013	1501	C: 96.3%[S: 49.7%, D: 46.6%], F: 2.1%, M: 1.6%, n: 954	Ref Table S1		SAMN14656765‐72	664	378	925	465	476
Chitonida	Chitonidae	*Acanthopleura loochooana*	China:Fujian	244,070,199	238,931	2462	C: 98.9%[S: 35.0%, D: 63.9%], F: 0.7%, M: 0.4%, n: 954	Ref Table S1		SAMN21190197‐202	461	243	632	241	293
Chitonida	Chaetopleuridae	*Chaetopleura apiculata*	USA: Massachusetts, Wood's Hole	68,204,335	72,499	1922	C: 81.8%[S: 74.4%, D: 7.4%], F: 11.0%, M: 7.2%, n: 954	Ref Table S1		SAMN14765620	967	720	1453	773	581
Chitonida	Chitonidae	*Chiton marmoratus*	British Virgin IslandS: St. Thomas	15,605,465	22,842	918	C: 29.7%[S: 27.7%, D: 2.0%], F: 12.5%, M: 57.8%, n: 954	Ref Table S1		SAMN14765613	210	314	439	254	132
Chitonida	Chitonidae	*Chiton tuberculatus*	British Virgin IslandS: St. Thomas	50,772,599	52,753	1983	C: 85.9%[S: 80.6%, D: 5.3%], F: 7.7%, M: 6.4%, n: 954	Ref Table S1		SAMN14765614	983	787	1546	898	751
Chitonida	Acanthochitonidae	*Choneplax lata*	Belize: Carrie Bow Bay	44,629,303	77,044	628	C: 12.2%[S: 10.8%, D: 1.4%], F: 16.2%, M: 71.6%, n: 954	Ref Table S1		SAMN16677971	182	235	335	175	79
Chitonida	Cryptoplacidae	*Cryptoplax japonica*	Japan	51,178,081	92,740	577	C: 29.4%[S: 27.7%, D: 1.7%], F: 29.0%, M: 41.6%, n: 954	Ref Table S1		SAMN16677973	373	517	740	381	234
Chitonida	Cryptoplacidae	*Cryptoplax larvaeformis*	Australia: Queensland, Heron Island	82,343,218	85,013	1920	C: 90.2%[S: 76.2%, D: 14.0%], F: 5.8%, M: 4.0%, n: 954	Ref Table S1		SAMN14765618	988	689	1441	749	494
Chitonida	Mopaliidae	*Katharina tunicata*	USA: Washington State, Friday Harbour, Cattle Point	46,724,187	54,149	1771	C: 93.3%[S: 84.0%, D: 9.3%], F: 3.8%, M: 2.9%, n: 954	Ref Table S1		SAMN14765625	1312	1342	2336	734	509
Chitonida	Ischnochitonidae	*Lepidozona mertensii*	USA: Washington State, Friday Harbour, Dead Man's Bay	38,585,755	55,242	1075	C: 72.5%[S: 64.2%, D: 8.3%], F: 9.9%, M: 17.6%, n: 954	Ref Table S1		SAMN14765626	672	721	1188	542	476
Chitonida	Chitonidae	*Liolophura sinensis*	Hong Kong: Kai Sai Chau	259,855,435	304,693	1559	C: 99.0%[S: 51.3%, D: 47.7%], F: 1.0%, M: 0.0%, n: 954	Ref Table S1		SAMN35319765‐69	555	339	790	270	316
Chitonida	Mopaliidae	*Mopalia muscosa*	USA: Washington, Friday Harbour, Cattle Point	36,547,733	45,808	1358	C: 80.0%[S: 71.8%, D: 8.2%], F: 9.7%, M: 10.3%, n: 954	Ref Table S1		SAMN14648874	954	1143	1830	611	390
Chitonida	Mopaliidae	*Nuttallochiton mirandus*	Antarctica: Weddell Sea	98,652,128	161,338	919	C: 76.2%[S: 64.9%, D: 11.3%], F: 13.1%, M: 10.7%, n: 954	Ref Table S1		SAMN14765623	765	658	1227	628	417
Chitonida	Schizochitonidae	*Schizochiton incisus*	China: South China Sea	21,828,186	25,671	1128	C: 39.0%[S: 35.6%, D: 3.4%], F: 20.2%, M: 40.8%, n: 954	Ref Table S1		SAMN32085629	847	491	1154	622	459
Chitonida	Ischnochitonidae	*Stenoplax bahamensis*	Belize: Carrie Bow Bay	80,786,861	119,802	810	C: 33.1%[S: 30.1%, D: 3.0%], F: 33.6%, M: 33.3%, n: 954	Ref Table S1		SAMN16677972	654	595	1059	547	433
Chitonida	Tonicellidae	*Tonicella lineata*	USA: Washington	222,614,494	357,556	1022	C: 97.4%[S: 54.5%, D: 42.9%], F: 2.0%, M: 0.6%, n: 954	Ref Table S1		SAMN14649070, SAMN08775006	1154	727	1713	446	338
Chitonida	Tonicellidae	*Boreochiton ruber*	Northern IrelanD: Kircubbin, Strangford Lough	112,487,450	150,734	1437	C: 77.3%[S: 65.7%, D: 11.6%], F: 11.0%, M: 11.7%, n: 954	Ref Table S1		SAMN41706136	1226	1028	1994	602	390
Chitonida	Chitonidae	*Tonicia schrammi*	USA: Florida, Panacea, Big Bend area	45,391,978	68,507	1106	C: 70.7%[S: 66.0%, D: 4.7%], F: 13.1%, M: 16.2%, n: 954	Ref Table S1		SAMN14765624	750	724	1283	663	543
Lepidopleurida	Hanleyidae	*Hanleya hanleyi*	Norway: Bergen	61,382,933	72,586	1577	C: 47.3%[S: 42.7%, D: 4.6%], F: 19.5%, M: 33.2%, n: 954	Ref Table S1		SAMN14765621	346	863	1029	466	261
Lepidopleurida	Leptochitonidae	*Lepidopleurus cajetanus*	Missing	48,866,159	174,781	300	C: 14.7%[S: 14.0%, D: 0.7%], F: 25.2%, M: 60.1%, n: 954	Ref Table S1		SAMN10483041	597	730	1156	598	296
Lepidopleurida	Leptochitonidae	*Leptochiton asellus*	Australia: Queens University Marine Lab; IrelanD: Ballyhenry Island	724,492,806	873,168	1367	C: 99.8%[S: 31.6%, D: 68.2%], F: 0.2%, M: 0.0%, n: 954	Ref Table S1		SAMN14765622, SAMN32105715‐19	327	222	508	123	98
Lepidopleurida	Leptochitonidae	*Leptochiton rugatus*	USA: Reid Rock, Friday Harbour, WA	57,855,285	79,070	1174	C: 80.3%[S: 61.5%, D: 18.8%], F: 12.4%, M: 7.3%, n: 954	Ref Table S1		SAMN03098848	721	1118	1642	690	428
Stylommatophora (Gastropoda)	Arionidae	*Arion vulgaris*	Germany	163,141,927	215,922	1090	C: 94.7%[S: 59.7%, D: 35.0%], F: 3.4%, M: 1.9%, n: 954	Ref Table S1		SAMN16874494	205	69	237	592	151

The genome assemblies were masked for repetitive regions and transposons using RepeatMasker v4.5.1 and RepeatModelor v2.0.4 (Tarailo‐Graovac and Chen 2009). For the RNA‐seq reads, paired reads with low‐quality bases (quality scores ≤ 7) covering more than 65% of the read length were filtered out. Then we merged the reads from the same species and used SPAdes v3.15.0 (Bankevich et al. 2012) for the transcriptome assembly (Table 1). Busco v5.5.0 was run in mode euk_tran to assess the completeness of genome assembly (−m geno) and the transcriptome assembly (−m euk_tran) using the lineage dataset metazoa_odb10 (Simão et al. 2015; Manni et al. 2021) (Table 1).

### 2.2 Identifying UCE Loci and Bait Design

#### 2.2.1 Genome‐Based Probe Set (GP)

The software PHYLUCE was used to identify UCE loci and design baits to target them using the online tutorial (Faircloth 2016). For the genome‐based probe (GP) design, *Acanthochitona discrepans* genome was used as the base reference genome (Sigwart et al. 2025), and two Gastropoda genomes, *Arion vulgaris* (Chen et al. 2022), and *Polymita picta* (Reyes‐Tur et al. 2025) were used as outgroups. We simulated sequencing reads from each assembly using ART v2.5.8 without inducing sequencing errors (Huang et al. 2012), and aligned the simulated reads to the reference assemblies using Samtools 1.19.1 (Li et al. 2009). The putatively orthologous loci (with a sequence divergence < 5%) shared among the reference and all chiton genomes (here also including *P. picta* ) were identified (Figure S1 and Table S2). Here, conserved loci were defined by loci shared by 90% of the species (the reference and other 16 species), which are 5756 loci (Figure S1 and Table S2). We merged loci with distances less than 100 bp, removed highly repetitive loci (loci with more than 25% repeats), and loci shorter than 160 bp, which remained 5681 conserved regions.

A temporary probe set was first designed to target those conserved regions from the reference genome. For each locus, we designed two baits with 3 times tiling that overlapped the middle of the targeted locus, and we removed potentially problematic bits with more than 25% of repeat content and GC content outside of the range of 30%–70%. All the baits were further aligned to themselves using LASTZ v1.04.03 (Harris 2007) to remove duplicates with the parameter “–identity 50 –coverage 50”, resulting in 11,308 probes.

The temporary probes were aligned to all genomes with an identity value of 50% and a minimum coverage of 83% (default value) for the master probe design. Loci captured by the temporary probes, ranged from 4726 loci (in one species) to 34 loci (shared by all the 20 species) (Table S2). We then extracted the loci which were present in at least 15 species as the ultimate UCE region, which is 3333 in total (Table S2). All the genomes were used for designing probes to capture these regions; the process and parameters were the same as for the temporary probe set design. After the removal of duplicates, we got 112,736 master probes. Finally, taking into account the costs of the probe set synthesis, we subset the master probe set to 9571 probes using probes from *Mopalia muscosa* , *Lepidozona retiporosa* , and *Polymita picta*. These representative species were randomly selected to represent ingroup and outgroup taxa, and *L. retiporosa* was deliberately included to maximise its restoration of UCEs.

#### 2.2.2 Transcriptome‐Based Probe Set (TP)

Transcriptome‐based probes (TP) followed the above approach with the following adjustmentS: *Acanthochitona rubrolineata* was used as the reference according to the completeness of BUSCOs, and *Arion vulgaris* as the outgroup (Table S1). Due to the low species occupancy of the transcriptomic UCEs in comparison with genomic UCEs, 75% of the species occupancy was assigned as conserved (shared by the reference and other 20 chitons), which is 8460 loci in total (Figure S1 and Table S3). After the removal of duplicated loci and probes, the temporary probe set has 16,685 probes, targeting 8372 conserved loci. The loci captured by temporary probes and shared by at least 15 species were further regarded as conserved loci, which is 2524 loci in total (Table S3). The master probe set designed by all transcriptomes contains 86,284 probes. We subset the master probe set to 10,552 probes using probes from *Leptochiton rugatus* , *Callochiton* sp., and *Katharina tunicata* .

#### 2.2.3 Mixed Probe Set (MP)

The genomic and transcriptomic probes were merged, and screened against each other to remove redundant probes using LASTZ v1.04.03 (Harris 2007) and BLAST v2.14.0 (Camacho et al. 2009) with an identity level of 50%.

### 2.3 *In Silico* Tests and Phylogeny

Probes designed for bivalves (BP) (González‐Delgado et al. 2024) and heterobranch gastropods (HP) (Moles and Giribet 2021) were additionally used to test their utility in resolving phylogenetic relationships of chitons. The five sets of probes: GP and TP were aligned to the chiton genomes and transcriptomes separately with an identity value of 50% using LASTZ v1.04.03 (Harris 2007), except for GP to genomes where we used a 75% identity value. MP, BP and HP were aligned to all the genomes and transcriptomes with an identity value of 50% (Table S4). To evaluate phylogenetic utility, we extracted loci targeted by the MP, including flanking regions extending 100, 200, 300, and 400 base pairs upstream and downstream of each locus. The sliced sequences were aligned to the probes again with a minimum coverage of 67% and a minimum identity of 80%, following the Phyluce tutorial (Faircloth 2016). For the other probe sets (GP and TP), the sequences were trimmed to include the core target regions along with 100 bp of downstream flanking sequence. Sequences that matched multiple probes, or where two or more sequences matched one probe were removed. Alignments of less than three species were removed.

Both coalescence‐based and concatenated phylogenetic trees were inferred from these alignments. For the coalescence‐based tree, unrooted gene trees were first constructed for each alignment using RAxML v8.2.12 (Stamatakis 2014) with 100 replicates with “‐m GTRGAMMA”. The best‐scoring tree was merged as input to ASTRAL v5.7.1, and the tree with branch length and bootstrap values was merged as input to weighted ASTRAL (wASTRAL) v1.19.3.7 to infer species tree separately with default parameters (Mirarab et al. 2014; Zhang et al. 2018; Zhang and Mirarab 2022). Branch support was assessed using local PP support (Sayyari and Mirarab 2016). We used the genesortR package to evaluate gene properties related to potential phylogenetic usefulness and bias for each locus with 100 base pairs upstream and downstream (Mongiardino Koch 2021), and by removing the top 1%, 10%, and 20% outlier genes, we generated three subset matrices. We reran wASTRAL on each subset to examine the impact on the phylogenetic trees.

For the concatenated tree, alignments with more than 75% of taxa (GP to genomes) and with more than 50% of taxa for all other matrices were selected, concatenated and aligned using MAFFT v7.453 (Katoh and Standley 2013). The resulting alignments were trimmed using GBlocks v9.91b with default parameters (Talavera and Castresana 2007) (Table S4). Maximum‐likelihood (ML) trees were inferred for each matrix using RAxML v8.2.12 (Stamatakis 2014) with the “‐m GTRGAMMA”, and bootstrap of 100; and IQ‐TEEE v2.1.3 (Minh et al. 2020) using ModelFinder (Kalyaanamoorthy et al. 2017) for model selection and 100 non‐parametric bootstrap replicates (−b 100).

## 3 Results

### 3.1 Probe Efficiency

The mixed non‐redundant chiton probe set (MP) consists of 19,020 probes, targeting 5730 UCE loci. MP comprises 9903 transcriptomic probes (TP) targeting 2459 loci and 9177 genomic probes (GP) targeting 3271 loci (Table 1). The GC content of TP and GP was slightly different, with TP (mean: 44.9%) being slightly higher than GP (mean: 44.3%) (Figure S2).

**FIGURE 1:**
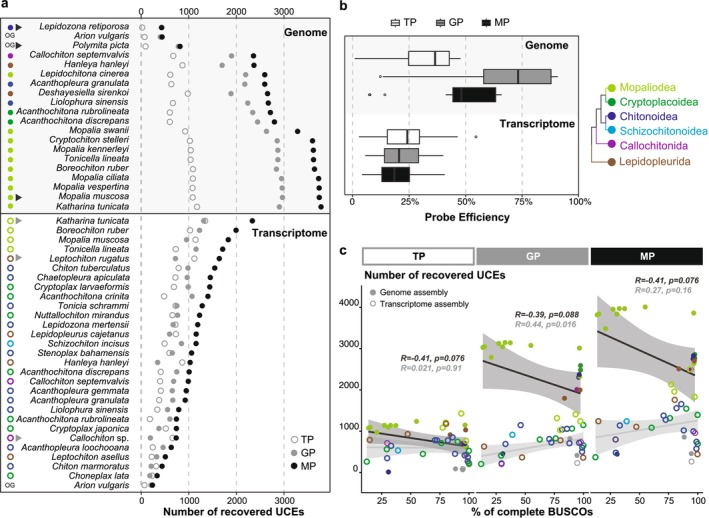
Performance of genome‐based probe set (GP), transcriptome‐based probe set (TP), and mixed probe set (MP). (a) Number of UCEs recovered by GP, TP, and MP across different species. Species indicated by triangles are those from which the total probe set was subsampled (see Methods). (b) Probe efficiency of GP, TP, and MP. (c) Relationship between the number of UCEs recovered by GP, TP, and MP and the completeness of the genome/transcriptome, measured by BUSCO completeness.

GP recovered an average of 2515 UCE loci in the 17 chiton genomes (max 2969 UCEs in *Mopalia muscosa* ; min 1701 UCEs in *Hanleya hanleyi* ), The average efficiency is 77%, except for *Lepidozona retiporosa* , which recovered 438 loci (Figure 1a,b and Table 1). Two gastropods, *Arion vulgaris and Polymita picta* recovered 401 and 780 loci, respectively (Figure 1a,b and Table 1). GP also yielded a considerable number of loci in transcriptome assemblies; an average of 702 UCEs per species were captured (max 1312 UCEs in *Katharina tunicata* ; min 182 UCEs from *Choneplax lata*), The average efficiency is 21% (Figure 1a,b and Table 1). A total of 2303 UCEs were captured by GP in at least three transcriptomes, which implies that at least 70% of the UCEs from genomic regions were located at genetic regions and actively expressed (Figure S3).

TP targeted an average of 612 UCE loci in the 28 chiton transcriptomes (max 1342 UCEs from *Katharina tunicata* ; min 184 UCEs from *Acanthochitona rubrolineata*), the average efficiency is 25% (Figure 1a,b and Table 1). In genomes, TP captured an average of 893 UCE loci per species, which is 1.5 times the number in transcriptomes (max 1174 UCEs from *K. tunicata* ; min 602 UCEs from *A. rubrolineata and A* . *discrepans* , except for *Lepidozona retiporosa* , which recovered 22 UCEs) (Figure 1a,b and Table 1).

MP recovered an average of 3148 UCEs from chiton genomes, which is on average 25% and 72% more than the loci obtained with genomic probes and transcriptomic probes only (max 3779 UCEs from *Katharina tunicata* ; min 2360 UCEs from *Callochiton septemvalvis* , *Lepidozona retiporosa* with 428 UCEs which were excluded from the counting) (Figure 1a and Table 1). In the transcriptomes, MP recovered an average of 1152 UCEs per species (max 2336 loci from *K. tunicata* ; min 335 loci from *Choneplax lata*), which is 1.6 and 1.8 times the loci obtained from the genomic and transcriptomic only (Figure 1a and Table 1). The average efficiency for the mixed probes is 63.6% in the genome and 23.3% in the transcriptome (Figure 1b). For each species, the UCEs recovered by the mixed probes are not simply the sum of the UCEs recovered by the genomic and transcriptomic probes separately; an average of 3.3% of the loci captured by transcriptomic and genomic probes separately could not be recovered using mixed probes due to the filtering out of multiple probes matching a single UCE and multiple UCEs matching a single probe (Figure S4). Five transcriptomic UCEs had not been captured separately before but were captured by the mixed probe set (Figure S4).

MP (contains 19,020 probes) is only one‐third of the Heterobranchia probes (HP: 57,606 probes), and comparable to the number of probes in the Bivalvia set (BP: 19,657 probes), The UCE loci recovered by MP were 2.67 and 3.58 times those recovered by HP (average 1181 UCEs) and BP (average 880 UCEs) from chiton genomes ( *Lepidozona retiporosa* was not included in counting), and 2.33 and 3.17 times those recovered by HP (average 494) and BP (average 363) from chiton transcriptomes (Table 1 and Figure S5a).

We found that the number of UCE loci captured in chiton genomes was not positively correlated with genome completeness evaluated by the completeness of BUSCOs, no matter TP (*r* = −0.41, *p* = 0.076), GP (*r* = −0.39; *p* = 0.088) or MP (*r* = −0.41; *p* = 0.076) (Figure 1c). However, the number of UCE loci captured in chiton transcriptomes was positively correlated with transcriptome integrity (Figure 1c). For HP and BP, the UCE recovered from both genomes and transcriptomes was slightly positively related to their completeness (Figure S5b).

### 3.2 Phylogenetic Performance of the Probe Sets

**FIGURE 2:**
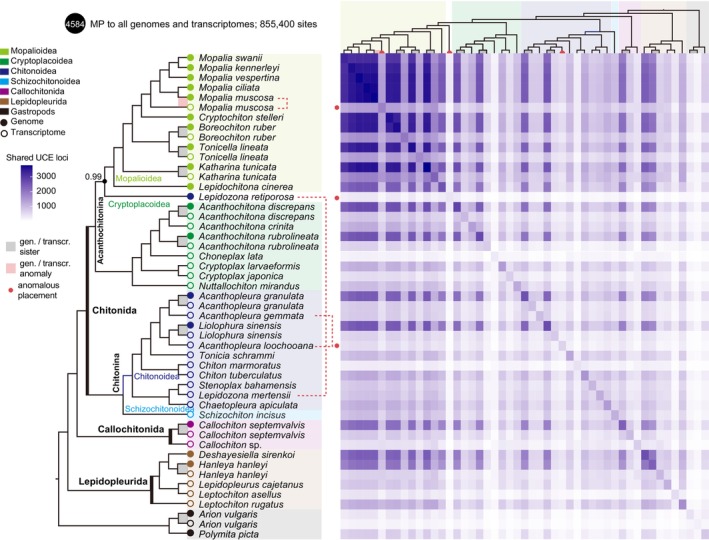
Performance of UCEs captured from mixed chiton probes (MP) in resolving phylogenetic relationships. Left: Phylogenetic reconstruction of all genomes and transcriptomes captured by MP. Species‐pairs connected by colour blocks—red for misplacement and grey for correct recovery; outlier species are marked with red dots following the species names. The red dashed line highlights where the species should be located. The tree is based on gene trees constructed using coalescent methods using wASTRAL; nodes with local posterior probabilities (Local PP) less than 1 are marked. Right: Heatmap showing the number of shared UCEs between each pair of species.

We compared results from the phylogenetic analyses based on the dataset recovered by the different probe sets to the expected phylogeny based on established data. We compared three main results in order to test the effectiveness of UCEs to recover a reliable phylogeny at different systematic levels: we tested data recovered by GP and TP probe sets on genome and transcriptome sequences separately (Figures S6–S9), and the MP, BP and HP on all available data (Figure 2 and Figures S6, S10–S12). While the backbone phylogeny for the group is robust, there are points of uncertainty at shallower depths (species and genus level) and these are discussed in more detail in the context of the main MP results (see Discussion).

#### 3.2.1 Phylogenetic Relationships Reconstructed From Genome Data

Both GP and TP targeted loci were able to recover the phylogeny from the genome data, except for *Lepidozona retiporosa*, which recovered far fewer sites than other chitons across different probes and in different matrices (Table 1; Figure 1a and Figure S7), with higher supports using the GP than the TP (Figure S8). The position of *L. retiporosa* was consistently anomalous in all phylogenetic analyses (Figures S8, S10, S12 and S13).

#### 3.2.2 Phylogenetic Relationships Reconstructed From Transcriptome Data

In the transcriptome data, GP recovered 2302 UCEs and TP recovered 1542 UCEs (Figure S6 and Table S4). Samples are underrepresented in most of the loci captured by both sets of probes, reflected in a significant reduction in the number of UCEs with more than 50% species coverage (by genomic probes: 250, an 89% reduction, and by transcriptomic probes: 533, a 65% reduction) (Figure S6 and Table S4). Both coalescence‐based species trees failed to recover the order‐level phylogenetic relationships (Figure S9). However, the 50% matrix species tree, although having very few loci, recovered order‐level topology, with higher bootstrap in transcriptome 50% matrix (Figure S9). At the genus level, there is one node with conflicts, with one tree failing to resolve *Acanthochitona* as monophyletic (Figure S9).

#### 3.2.3 Phylogenetic Relationships Reconstructed From Combined Data

The mixed probe set recovered 4584 UCEs in all the genomes and transcriptomes, including 855,400 informative sites (Figures S6 and S7; Table S4). We obtained a phylogenetic tree that includes all chitons with available genomic and transcriptomic data and is well resolved at both the order level, family level, and even species level (Figure 2). Ten out of eleven genome‐transcriptome pairs of the same species were clustered together, except for *Mopalia muscosa* (Figure 2). The genomic and transcriptomic data for *M. muscosa* were produced by separate efforts; the genome lacks information on the collection site, while the transcriptomic individual is from Friday Harbour, USA (Table 1).

The concatenated tree constructed from the 50% occupancy matrix (1316 UCE, 311,030 informative sites) recovered deep relationships, but showed weaknesses in relationships between transcriptome/genome data in the same and closely related species (especially in Mopaliodea, Figure S10). Species with the same data source (transcriptome or genome) are more likely to cluster together.

#### 3.2.4 Performance of Heterobranch Gastropod and Bivalve UCE Probes

HP targeted an average of 1181 UCEs in chiton genomes (excluding the problematic *Lepidozona retiporosa* , 249), and 494 UCEs in chiton transcriptomes (Figure S11). BP targeted an average of 880 UCEs in chiton genomes and 363 UCEs in chiton transcriptomes (Figure S11). Both recovered the relationship among the main clades (Figure S12), except the tree reconstructed using all UCEs from the bivalve probes, clustering *Callochiton septemvalvis* with members of the Acanthochitonina clade (Figure S12). However, within each order or superfamily, many relationships are in conflict with each other and many nodes are poorly supported (Figure S12).

#### 3.2.5 Influence of Reconstruction Methods and Flanking Regions

**FIGURE 3:**
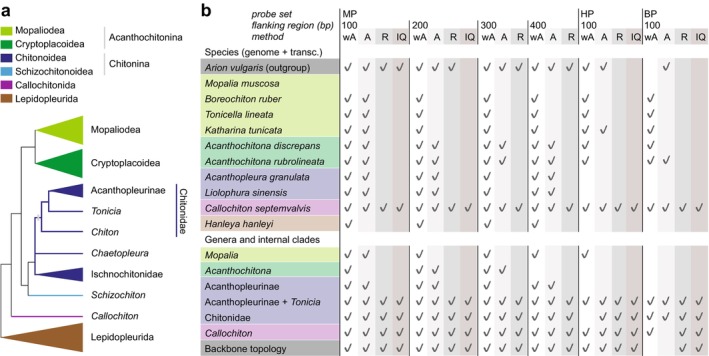
Illustration of the phylogenetic success of the UCE analyses compared to the expected topology. (a) Schematic phylogeny of sampled polyplacophoran molluscs, illustrating major clades checked in comparative analyses. (b) Nodes examined in comparative analyses using three probe setS: Mixed chiton probe set (MP), bivalve probe set (BP), and heterobranch gastropod probe set (HP), with different size flanking regions retained (number in bp) under three different analytical methodS: WASTRAL (wA), ASTRAL (A), RaxML (R), and IQTree (IQ). Tick marks indicate taxon groups that were recovered with the expected topology, for species pairs as sister lineages (where a single species is represented in the analyses by separate data from genome and transcriptome), and genera or wider clades as monophyletic, as illustrated on the schematic tree.

Another important factor in the design is the size of the selected flanking sequences of the UCEs. We tested phylogenetic reconstructions based on 100, 200, 300, and 400 bp flanking regions using MP on all data. Increasing flanking regions did not improve phylogenetic resolution (Figure 3 and Figure S13). Larger flanking regions exacerbated the separation of transcriptome‐ and genome‐based data, so that more species “pairs” resolved incorrectly. The decrease in resolution was not a direct inverse relationship as the flanking region increased: the analyses with flanking regions of 300 bp performed more poorly than 400 bp.

Among the reconstruction methods compared, coalescent methods (ASTRAL and wASTEAL) outperformed concatenated methods (RaxML and IQTree) in producing well‐resolved trees (Figure 3). Coalescent methods were the only approach that successfully recovered sister‐group relationships in transcriptome‐genome pairs from the same species (Figure 3 and Figure S13). Moreover, wASTRAL outperformed Astral, successfully reconstructing the sister relationships of the *Acanthochitona* and *Hanleya* genome‐transcriptome pairs. RaxML and IQTree analyses recovered deeper phylogenetic relationships but failed to resolve many families to genus‐level relationships, as both methods tend to cluster species with similar data types together—which is particularly noticeable within the Mopaliodea clade (Figure S13). The one exception to this general pattern was that data based on the bivalve probe set performed less well with coalescent methods than with the other methods; this was the only case in which the relationships among orders (Callochitonida versus other chitons) were incorrectly recovered (Figure 3 and Figure S12).

#### 3.2.6 Loci Properties and Influence of Phylogenomic Subsampling

Phylogenomic subsampling has been widely used to reduce data heterogeneity, improve model fit, and test the robustness of phylogenetic inference (Mongiardino Koch 2021). Following this rationale, we evaluated the characteristics of loci captured by our probes (Figure S14). We found that loci captured with TP showed slightly higher root‐to‐tip variance, evolutionary rate, and saturation compared to those captured with GP. In contrast, loci captured by GP had higher occupancy and longer alignment length. Nevertheless, both datasets followed consistent overall trends and levels. To test the influence of phylogenomic subsampling, we reconstructed phylogenies using matrices that filtered out 1%, 10%, and 20% of outlier loci. In all cases, the same relationships were recovered; only two nodes showed a minor reduction in local posterior probability (from 1 to 0.99) under the 10% and 20% filtering schemes (Figure S15). These results further highlight the robustness of UCEs for phylogenetic inference in different matrices in chitons.

## 4 Discussion

### 4.1 Utility of UCEs in Data‐Limited Clades

Polyplacophoran molluscs offer several important advantages as a test case, and cover multiple scenarios that other under‐studied organisms will likely represent. The backbone phylogeny here is well understood, but the total clade species richness is high and encompasses species radiations at different depths and rates. Much of the available taxon sampling covers species assemblages that are well studied in terms of adaptations (e.g., Varney et al. 2021) or phylogeography (Kelly and Eernisse 2008), as well as additional data gathered by many different researchers working opportunistically or deliberately to fill taxonomic gaps (e.g., Liu et al. 2023). This foundation and a mixture of available data provide an opportunity to test the influence of probe design, source data, and phylogenetic inference methods to optimise future studies to expand taxon sampling with de novo sequencing.

In our *in silico* tests, loci captured by probes designed from either transcriptomes or genomes exhibited relatively consistent and stable characteristics as well as phylogenetic informativeness, demonstrating their potential for recovering evolutionary relationships. Our results indicate that applying different levels of filtering did not affect tree topology with the coalescent method, even when one‐fifth of the loci were excluded. However, our results highlight the importance of both tree‐constructing methods and the choice of flanking regions. If there are enough molecular markers and they are evenly distributed, both the gene tree and species tree can reproduce the same relationships. If the markers are not evenly distributed among species, there will be serious discrepancies. In our results, the impact of tree‐building methods is substantially greater in transcriptomic data than in genomic data. For instance, species trees inferred using the coalescent method with all available loci failed to recover ordinal‐level phylogenetic relationships in transcriptomic datasets, regardless of whether GP or TP were used. However, after applying a 50% matrix occupancy filter, despite the significant reduction in the number of loci, deeper phylogenetic relationships were more accurately recovered using the concatenated method (Figure S9). Within the genomic datasets, GP and TP generally showed comparable performance. Using MP in the combined genome and transcriptome datasets, a coalescent‐based approach appears more effective at reducing the impact of large differences in locus sharing among species and the influence of missing data, thereby recovering more accurate relationships with short divergent times. In our results, the optimal combination was 100 bp flanking regions analyzed with wASTRAL, which successfully resolved phylogenetic relationships across all target levels, from deep to shallow divergences (Figure 2). This is likely to also be the case in any study combining newly sequenced data and previously published sources to increase taxon sampling.

We anticipated that expanding flanking sequences would more sensitively reflect recently divergent (species‐level) relationships, while the reduction of flanking sequences would more stably restore deep species relationships. The *in silico* tests provided confounding evidence to this hypothesis, indicating that the reduced dataset with a smaller flanking region produced more stable and more reliable topologies. This provides a guide for future studies, but each dataset may require tailored adjustment, noting that such adjustments become more challenging when there is no strong a priori phylogenetic hypothesis. For example, if higher species coverage (more conservative sites) is the priority, more flanking sequences can be truncated to recover the phylogenetic relationships of the species.

Our analyses are based entirely on *in silico* simulations. We recognize that empirical performance can be influenced by factors such as DNA quality, hybridization efficiency, and off‐target noise, particularly when working with historical museum samples. However, the large number of loci captured using our probe set provides a measure of confidence, and insurance, that future applications will obtain a sufficient quantity of loci. This is an essential finding to enable and justify future applications to perform target‐enrichment sequencing of museum specimens and to integrate the growing body of genomic and transcriptomic resources in order to resolve the complete chiton phylogeny. At the same time, considering the varying scales of sampling in empirical applications and the need to resolve phylogenetic relationships across different evolutionary timescales, we encourage future studies to explore a range of parameter settings and analytical methods to assess topological stability and to recover phylogenies that most closely approximate true evolutionary history.

The tests of previously published bivalve and heterobranch gastropod probe sets on a different class of molluscs further confirm the general usefulness of UCEs at deep phylogenetic levels. Chitons diverged from Conchifera (the clade including Bivalvia and Gastropoda) an estimated 548 million years ago, and the divergence among different chiton orders occurred approximately 200–300 million years ago (Chen et al. 2025). Our results show that, although probes designed for bivalves and gastropods were able to capture a subset of loci in chitons and resolve ordinal‐level phylogenetic relationships, they were insufficient for resolving relationships at shallower taxonomic levels. The successful application of the new probe set presented here underlines the importance of custom‐designed probe sets especially for recovering shallow, species‐level relationships.

### 4.2 Phylogeny of Polyplacophora

The present analysis primarily used available genome and transcriptome data for chitons from previous studies. The majority of available data is based on transcriptomes, as relatively few genomes are available for this large group. In addition to the limited taxon sampling, the available data offer uneven quality. A lack of comparative data also can lead to inadvertent quality issues with potential misidentification or contamination that ultimately result in anomalous phylogenetic positions. There is room for further improvement, but the impact does not significantly impede good phylogenetic resolution when interpreted critically.

All analyses resolved the expected topology and membership of the major groups within Polyplacophora: Lepidopleurida is the earliest divergent order, sister to all other chitons, followed by *Callochiton*. The clade Chitonida comprising all other species is divided into two suborders, Chitonina and Acanthochitonina. *Schizochiton* is sister to all other Chitonina (Sirenko 2006; Liu et al. 2023). Acanthochitonina is divided into two superfamilieS: Cryptoplacoidea and Mopalioidea (Okusu et al. 2003; Sirenko 2006). The position of sampled taxa provides insights both for the evolution of chitons and also the performance of UCE‐based analyses.

Within Mopalioidea, the main analysis recovered the genus *Mopalia* but with different ingroup relationships compared to previous work (Kelly and Eernisse 2008). The positions of *Cryptochion stelleri* and Tonicellidae (*Tonicella* + *Boreochiton*) follow previous results (Sigwart et al. 2013; Irisarri et al. 2014). The position of *Katharina* outside of Mopaliidae (*Mopalia* + *Cryptochiton*) is unexpected but not implausible; and likewise, *Lepidochitona* would be expected to resolve within Tonicellidae but the systematics within this clade are expected to require revision.

Cryptoplacoidea are represented here by a few distinct genera covering three families. In the transcriptome‐based tree (Figure S9) *Acanthochitona* is recovered paraphyletic with respect to *Choneplax lata*, with low support. The main analyses of MP to all genomes and transcriptomes recovered the genus *Acanthochitona* as monophyletic, but only wASTRAL recovered the expected ingroup relationship: *A. crinita* was recovered as sister to *A. discrepans* , whereas other methods recovered *A. crinita* sister to *A. discrepans* + *A. rubrolineata* (Figure 2 and Figure S13). *Choneplax lata* was recovered as sister to *Acanthochitona*, consistent with previous analyses (Irisarri et al. 2014; Irisarri et al. 2020; Liu et al. 2023). *Cryptoplax larvaeformis* and *Cryptoplax japonica*, formed a clade that was sister to the A*canthochitona* + *Choneplax* grouping. *Nuttallochiton* was recovered as sister lineage to other Cryptoplacoidea, in agreement with prior molecular studies (Sigwart et al. 2013; Irisarri et al. 2014; Irisarri et al. 2020; Liu et al. 2023).

The sampled species in Chitonina focus on species with sensory adaptations in shell eyes (Acanthopleurinae + *Tonicia*) and eyespots (*Chiton* spp.) which form a clade in a derived position (Liu et al. 2023). The remaining sampled Chitonina form a clade including Ischnochitonidae (*Lepidozona* + *Stenoplax*) sister to *Chaetopleura* (Chaetopleuridae). Although the analyses recovered the expected topology, one persistent anomaly within the Acanthopleurinae is the sister‐group relationship of *Acanthopleura loochooana* and *L. sinensis* , resulting in a paraphyletic genus *Acanthopleura*. Other analyses found a paraphyletic *Acanthopleura* based on the same source data (Owada 2023) or mitochondrial genomes including *A. loochooana* and other congeners (Alnashiri et al. 2024; Kim et al. 2024), and a larger study recovered *A. loochooana* in the genus *Squamopleura* (Alnashiri et al. 2023). There is ongoing taxonomic uncertainty in this subfamily Acanthopleurinae (Ibáñez and Sirenko 2024). At the same time, this group is prone to misidentification and validating identifications requires sequence data connected to voucher specimens or high‐quality photographs.

Lepidopleurida remains an enigmatic group within chitons; as in previous analyses *Lepidopleurus cajetanus* and *Leptochiton asellus* resolve as sister species, representing the family Leptochitonidae *sensu stricto* (Sigwart et al. 2010). Previous analyses recovered a similar topology with Protochitonidae (*Deshayesiella*) sister to Leptochitonidae *s.s*. (Sigwart et al. 2010; Sigwart 2017). The position of *Hanleya* was not stable in those earlier studies, which found it either nested within Lepidopleurida (Sigwart et al. 2010) or as a basal branching lineage sister to the rest of the clade (Sigwart 2017), and other phylogenomic studies have insufficient taxon sampling for this issue (Kocot et al. 2011; Chen et al. 2025), so the question remains open.

The phylogenetic results here highlight a number of open questions in chiton systematics, especially for the topology within Mopalioidea, the membership of *Acanthopleura*, and the position of *Hanleya*, all of which have been noted in previous literature with other methods. New data are required to resolve these questions but the application of UCEs to increase sampling offers a promising way forward.

### 4.3 UCEs Improve Phylogenetic Resolution for Understudied Groups

Selecting appropriate molecular markers is crucial when constructing phylogenetic trees at the molecular level. Phylogenies reconstructed from mitochondrial genes and genomes are widely accepted as not reflecting nuclear gene phylogenies, and this has been confirmed repeatedly in different metazoan groups (Nosenko et al. 2013; Morgan et al. 2014; Platt et al. 2018; Jousselin et al. 2024). The number of orthologous gene sets that can be selected and used as molecular markers from genome and transcriptome data depends fundamentally on the completeness of available sequencing and assembly. In molluscs, as in many other organisms, there are practical as well as bioinformatic barriers to acquiring high quality sequence data (Sigwart et al. 2021; Chen et al. 2025).

Single‐copy orthologous genes are considered the ideal tools for phylogenetic inference but high‐quality genomic data not only require a high standard for the preservation status and time of the sample, i.e., the quality of the DNA, but also the time and monetary investment of sequencing, assembly and annotation. These costs are greatly increased in complex genomes of under‐studied animals where there is no strong a priori basis to estimate the expenses (Chen et al. 2025). In comparison to genomes, transcriptomes have a relatively simple process, and this leads to relatively larger scale species coverage as seen here. However, this represents a clear trade‐off as the number of genes is lower in transcriptome data, and the number of orthologous genes will decrease further with the increasing number and diversity of species included. Datasets that differ in the quantity and distribution of missing data and species coverage result in different topologies, which represent a perennial issue in phylogenetics (Sanderson et al. 2010; Nabhan and Sarkar 2012).

In chitons, as in many large clades, rare and/or otherwise inaccessible species represent a large fraction of diversity, with many known only from a handful of specimens or observations. But these rare species represent key lineages that can have a disproportionate influence on the topology in phylogenetic analyses. One of the great hopes for UCE‐based methods is that these techniques are suitable for material with degraded or fragmented DNA (McCormack et al. 2016; Derkarabetian et al. 2019), which is the typical state of material preserved in museums for over 100 years in dry or ethanol‐preserved conditions. UCE approaches are rapidly increasing in importance in phylogenomics.

We expected that the large differences in the number of UCE loci could lead to biased phylogenetic inferences. Coalescent‐based tree approaches seemed to be relatively robust to this. As expected, transcriptome‐based data resulted in fewer UCEs than genome‐based data; these differences are most clear in the species pairs, and may be an important aspect in the anomalous results when these pairs do not resolve as sister taxa. Transcriptome data are predominantly exons, whereas in general most UCEs are non‐coding and found in introns, intergenic regions, and untranslated regions (Polychronopoulos et al. 2017). The subset of exonic UCEs is mainly from regulatory genes (Sandelin et al. 2004; Woolfe et al. 2004; Elgar and Vavouri 2008). While these are undoubtedly homologous regions, it does introduce potential bias, and evidentially the transcriptome‐based data resolve less stably within a combined phylogeny. It is also interesting that the mixed probe set design produced the best overall performance, so the inclusion of transcriptome data is clearly beneficial.

Most projects working on increasing phylogenetic resolution will want to make use of all possible available data, and so these limitations on combining data harvested from previously transcriptomes are an important caution for future work. Our probes can be used not only for targeted enrichment sequencing of museum chiton samples in the future, but also as molecular markers that can be utilised to construct a phylogeny by integrating all the new genomic and transcriptomic resources of chitons in the future, applied with scrutiny especially to the placement of transcriptome‐based data.

At broader taxonomic scales, it is also important to note the separation among the results from probe sets optimized for different groups within molluscs. Combinations of these different probe sets should provide effective tools to sample across the phylum and more broadly. We hope that they can be used as a tool to correct the confusing classification. Furthermore, studying the important roles and functions played by these UCEs in molluscs will further our understanding of species evolution.

## Author Contributions

Conceptualization: Z.C., K.V., J.D.S. Data curation: Z.C. Formal analysis: Z.C. Funding acquisition: J.D.S. Investigation: Z.C., K.V., J.D.S. Methodology: Z.C. Supervision: J.D.S. Validation: Z.C., K.V., J.D.S. Visualisation: Z.C., J.D.S. Writing – original draft preparation: Z.C., J.D.S. Writing – review and editing: Z.C., K.V., J.D.S.

## Conflicts of Interest

The authors declare no conflicts of interest.

## Supporting information


**Data S1:** men70076‐sup‐0001‐supinfo.docx.

## Data Availability

The probe sequences were deposited in Figshare: Chen, Zeyuan; Vončina, Katarzyna; Sigwart et al. (2025). Chiton probe sets to capture Ultralconserved Elements (UCE). figshare. Dataset https://doi.org/10.6084/m9.figshare.28921088.v1.
