# Supplementary material for: Evaluating Probe Design for Phylogenomics Across Taxonomic Scales: First Steps for Applying Ultraconserved Elements in an Understudied Class (Mollusca: Polyplacophora)
Source: Mol Ecol Resour. 2025 Nov 13;26(1):e70076. doi: 10.1111/1755-0998.70076 (PMC12614043; doi:10.1111/1755-0998.70076)
Supplement: Supplementary file 1 — Data S1: men70076‐sup‐0001‐supinfo.docx. [file MEN-26-e70076-s001.docx]

**Supplementary Figures and Tables**

**Evaluating probe design for phylogenomics across taxonomic scales: First steps for applying ultraconserved elements in an understudied class (Mollusca: Polyplacophora)**

Zeyuan Chen, Katarzyna Vončina, Julia D. Sigwart


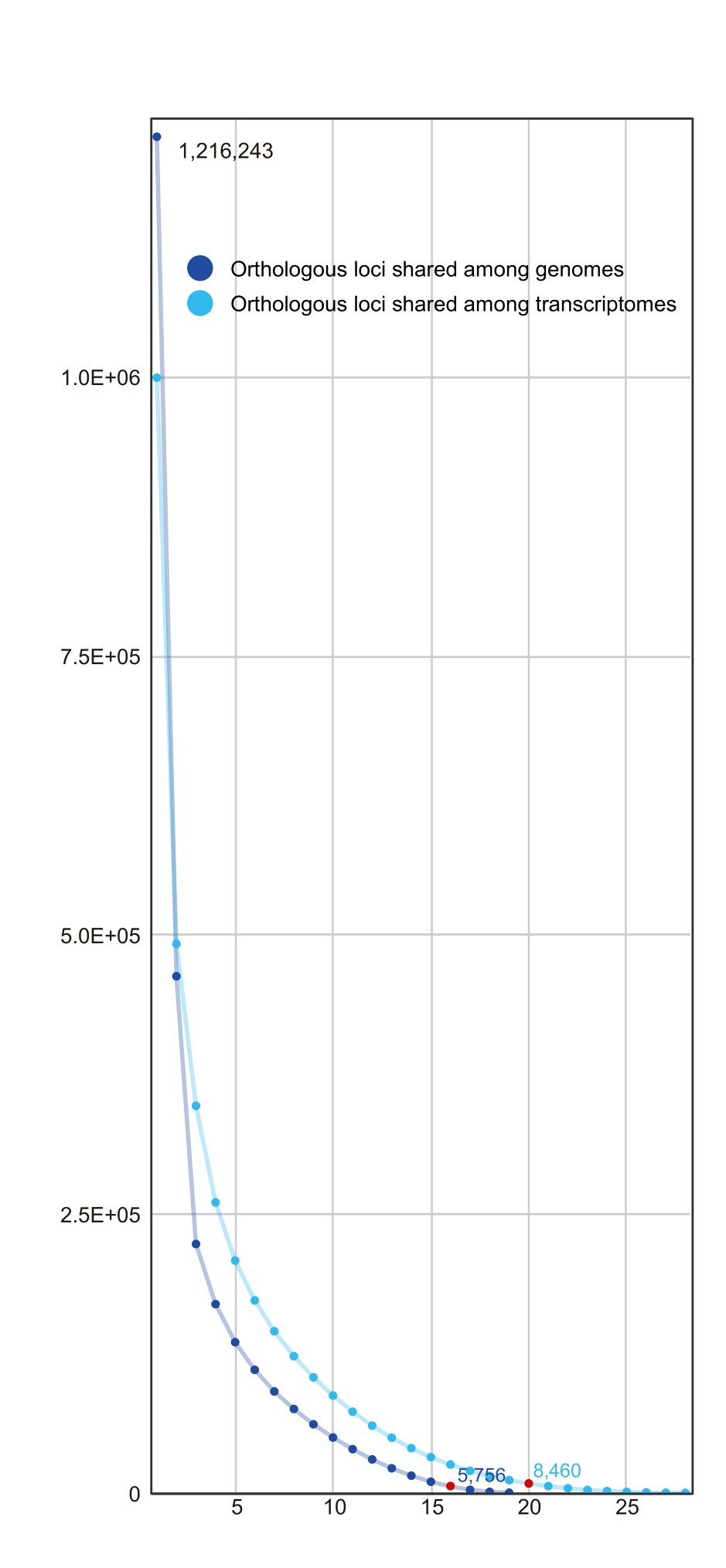


**Figure S1** Number of conserved loci shared among assemblies among the reference and other chiton genomes/transcriptomes.


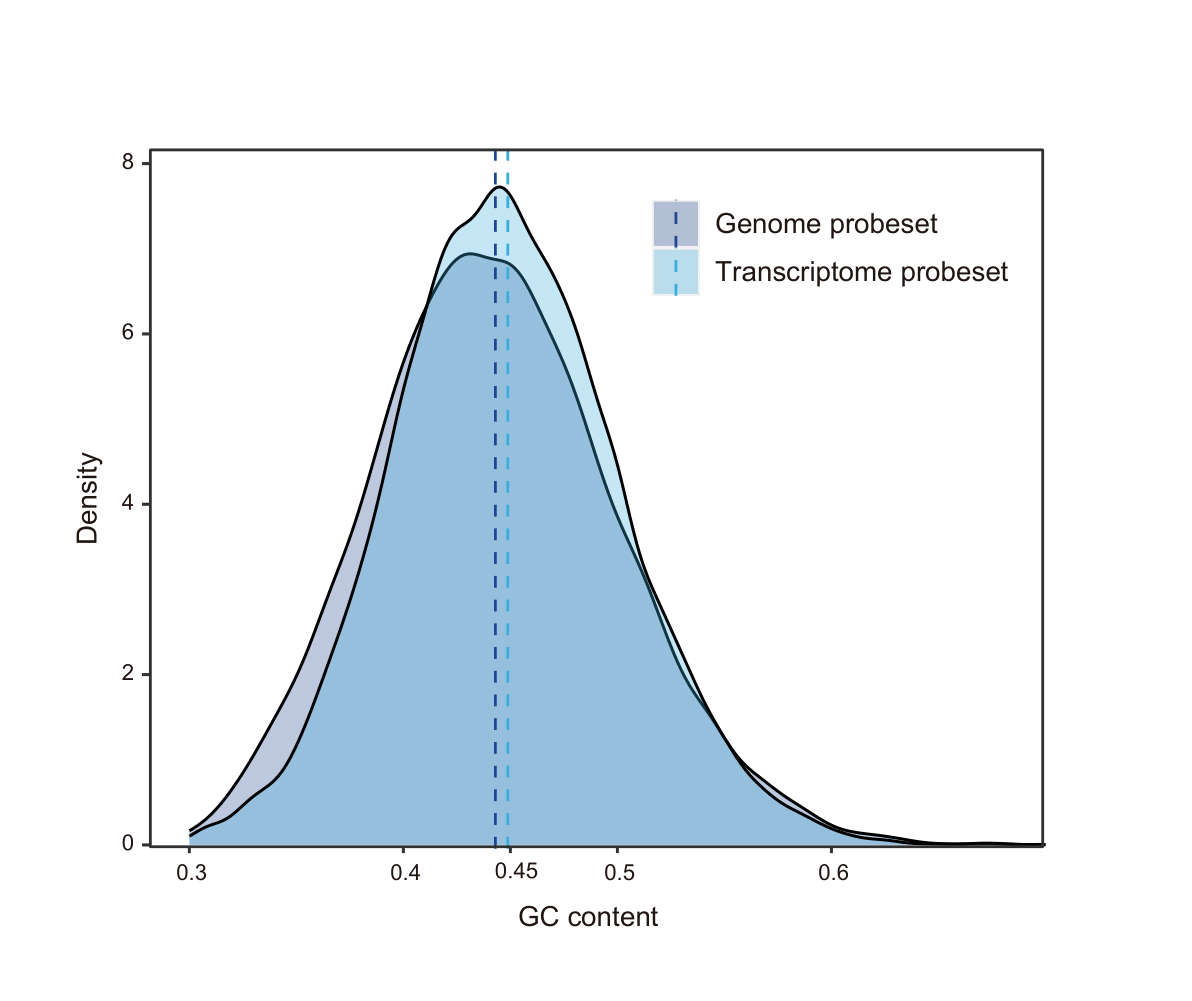


**Figure S2** GC content of genomic and transcriptomic probe set.


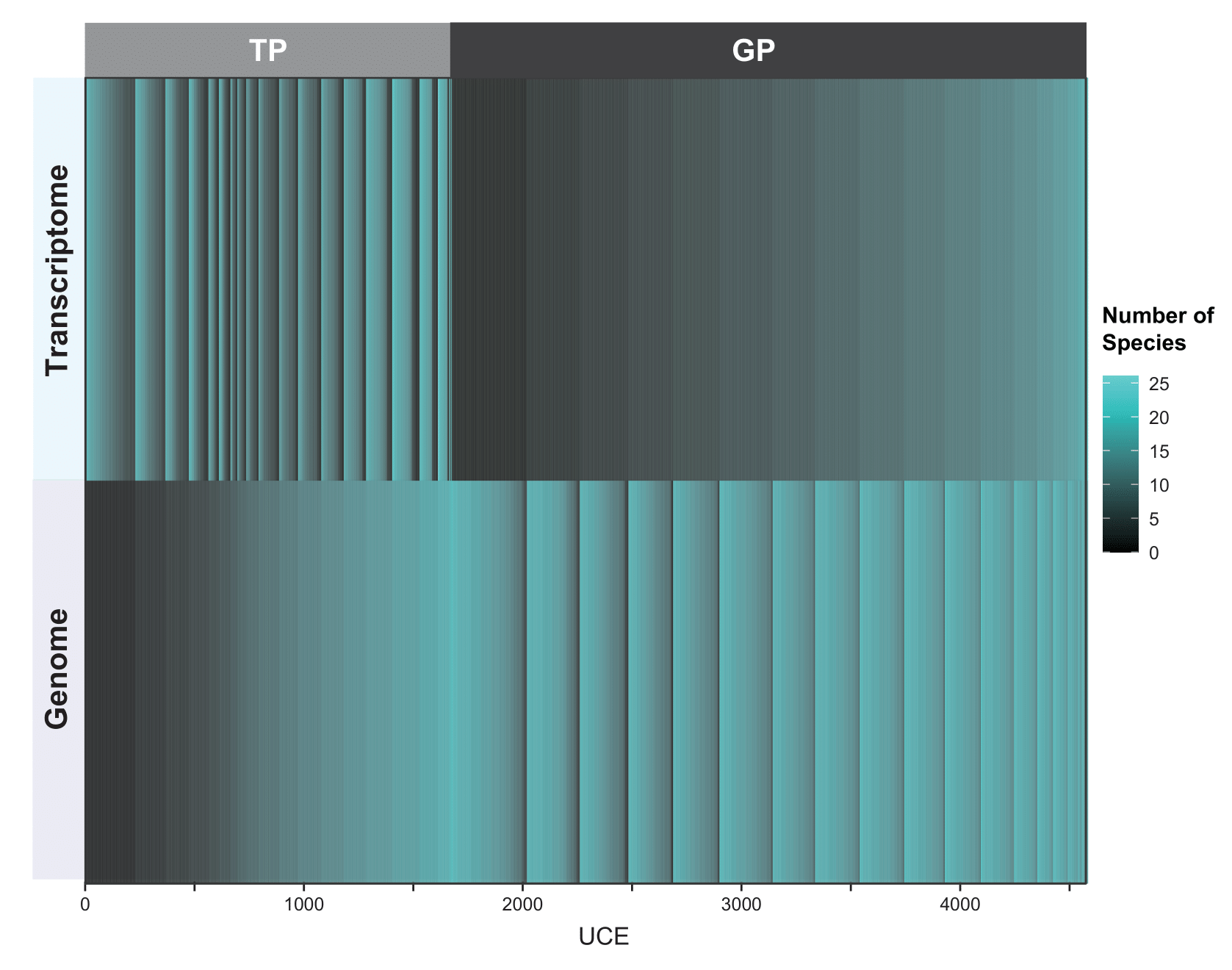


**Figure S3** Species occupancy in each UCE recovered using Transcriptomic (TP) and Genomic (GP) probes.


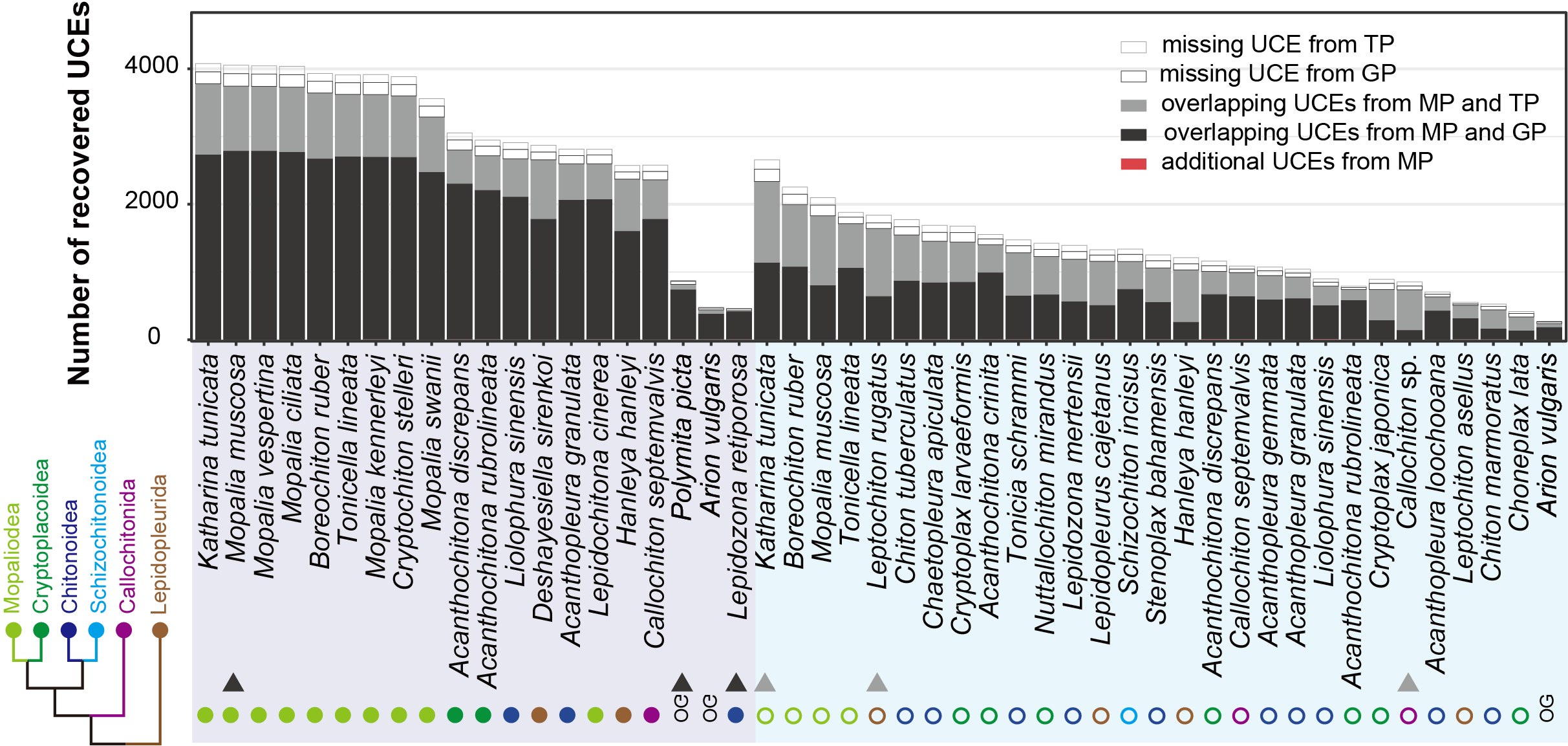


**Figure S4** Composition of UCEs recovered by the mixed probe set in each species. The species pointed to by the triangles are the species used when taking a subset of probes. GP: genome-based probes, TP: transcriptome-based probes, MP: mixed probes. Species names with a dark blue background (left side) indicate genome data, while those with a light blue background (right side) represent transcriptome data.


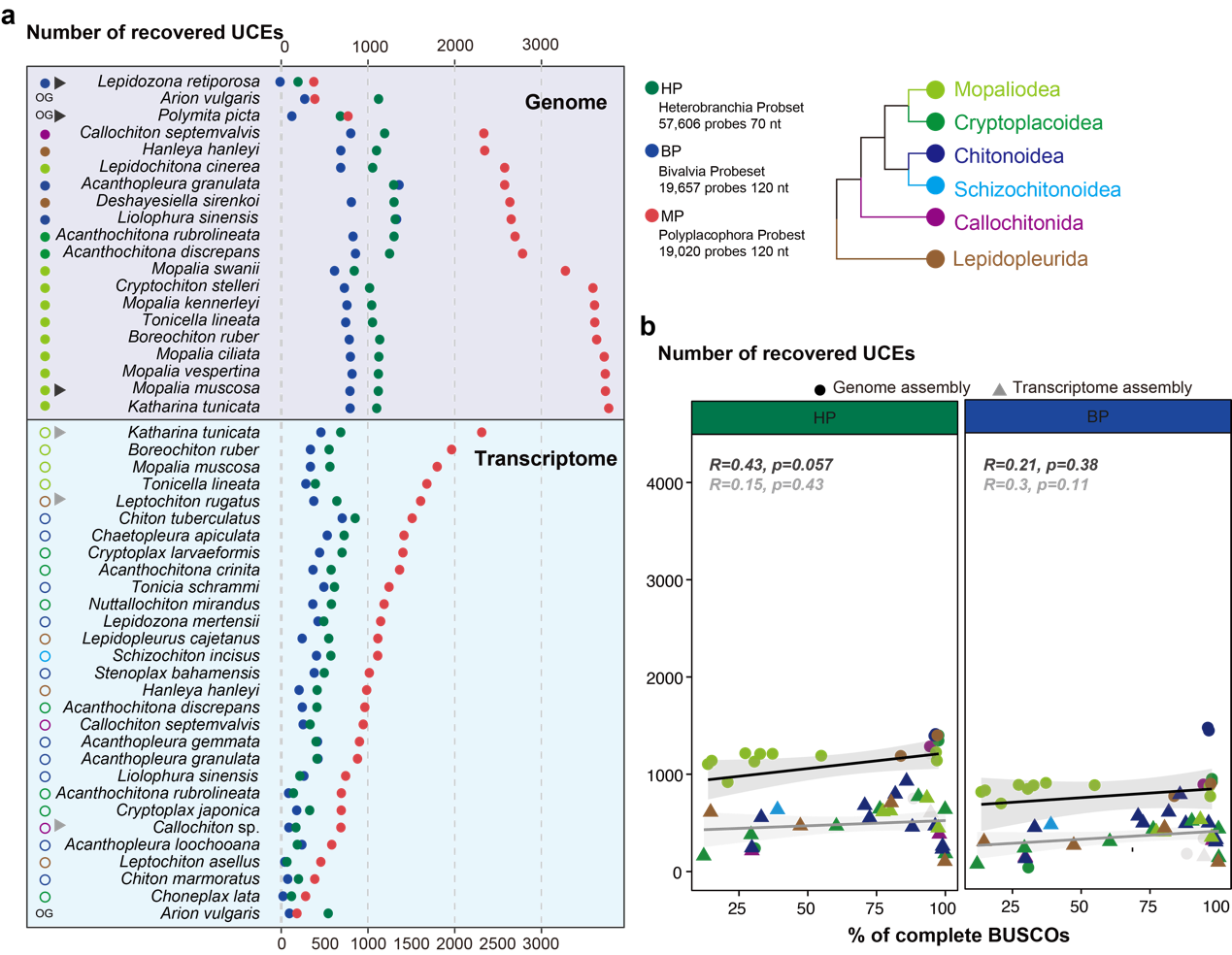


**Figure S5 a)** Comparison of UCE loci recovered with Gastropoda (Heterobranchia, HP) probes and Bivalvia probes (BP) and mixed chiton probes (MP) in chiton genomes and transcriptomes. **b)** Correlation of UCE loci recovered using Gastropoda (Heterobranchia, HP) probes and Bivalvia probes (BP) with BUSCO completeness.

**
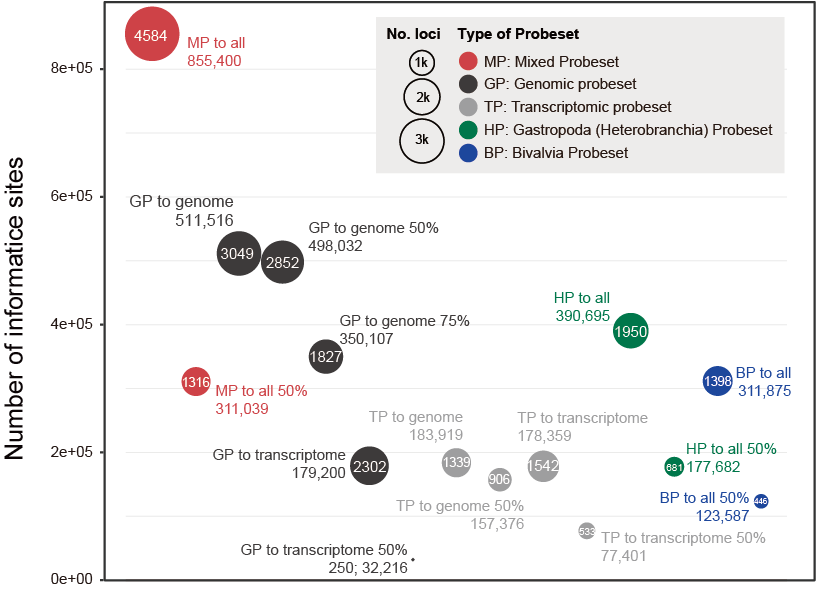
**

**Figure S6** Total number of UCE loci with phylogenetic information (number of shared species greater than or equal to three) captured by different probes, and number of informative sites. GP: genome-based probes, TP: transcriptome-based probes, MP: mixed probes. HP: Gastropoda (Heterobranchia) probes, BP: Bivalvia probes.


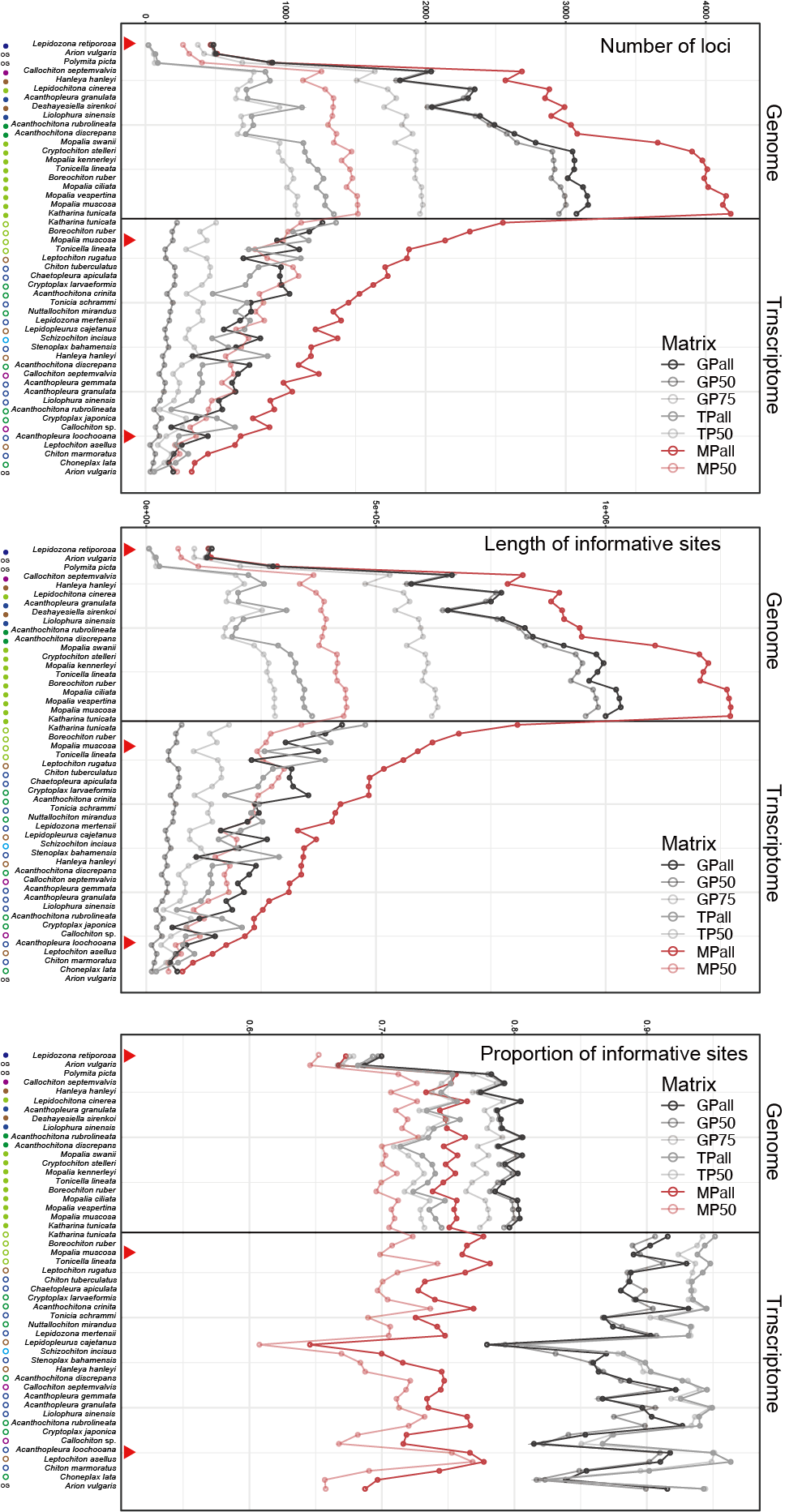


**Figure S7** Number of UCE loci, cumulative informative length, and proportion of informative sites (calculated as the proportion of non- “-” sites over the total sites) per species in phylogenetic matrices of different occupancy levels for GP (Genome-based), TP (Transcriptome-based), and MP (Mixed) probe sets.


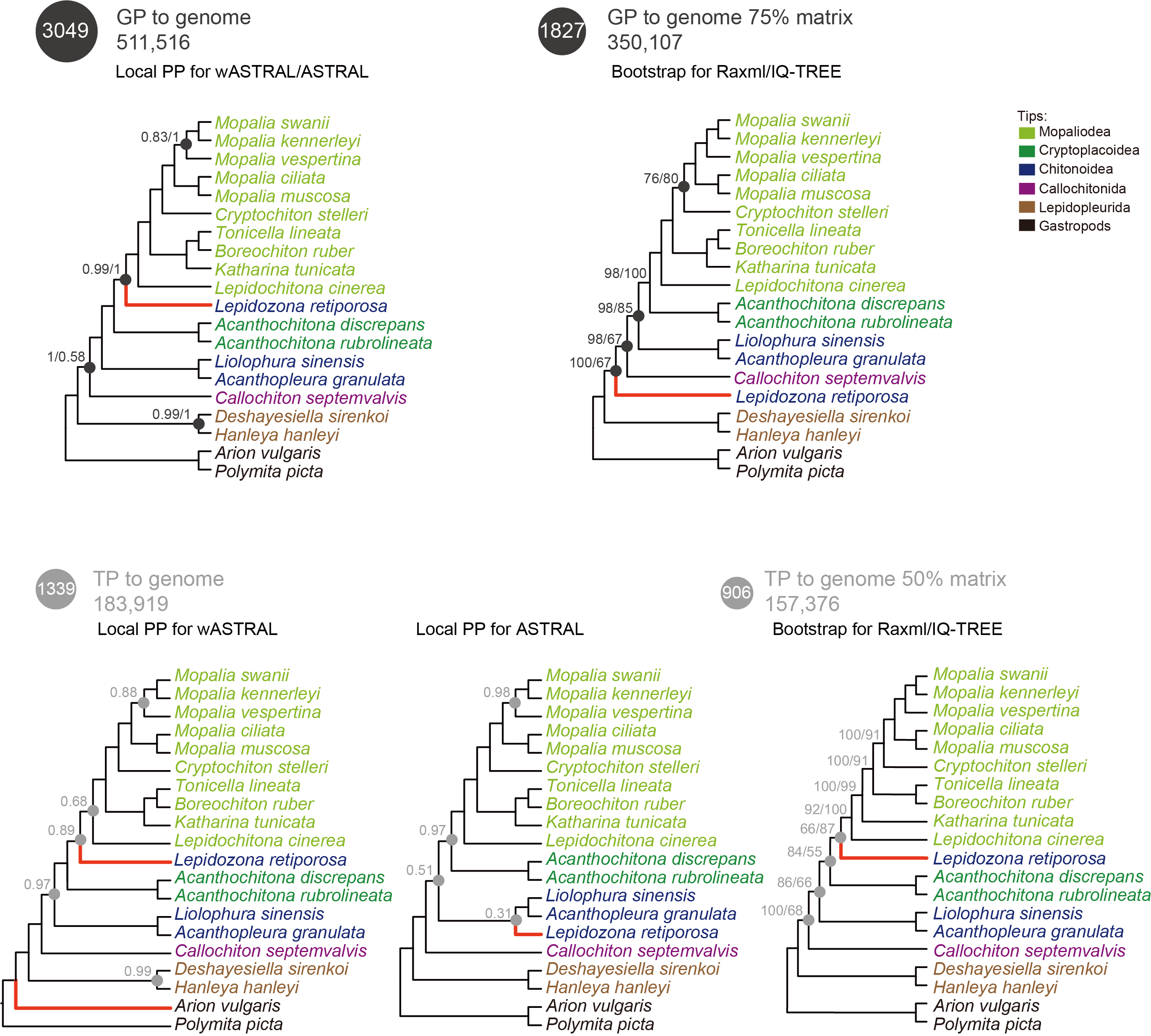


**Figure S8** In-silico test and topology of the Genome-based probes (GP) and transcriptome-based probes (TP) on chiton genomes. Branches that conflict with taxonomic expertise (and may indicate potential issues) are highlighted in red. Nodes without full local posterior probabilities (Local PP) or bootstrap values are labeled accordingly.


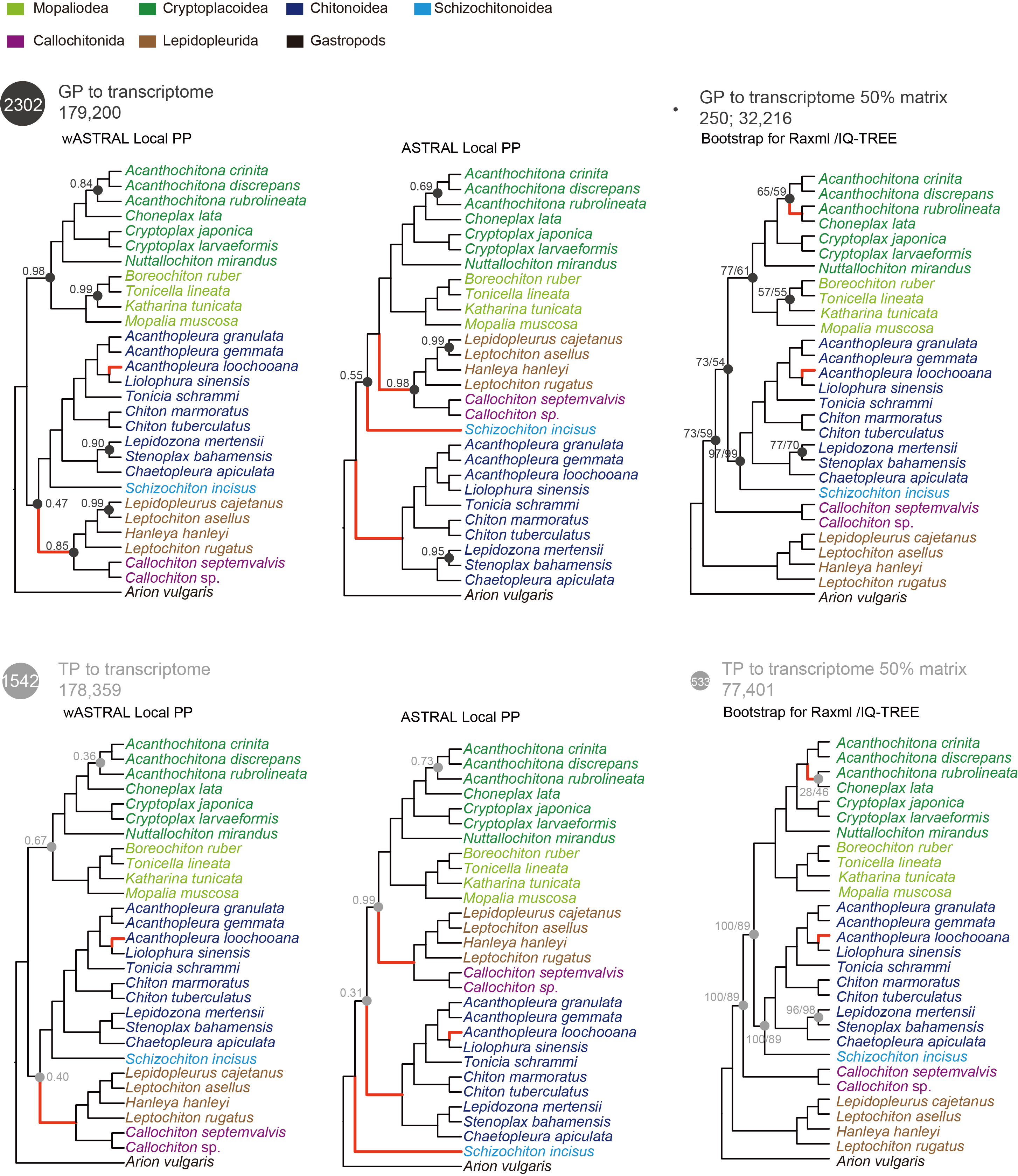


**Figure S9** In-silico test and topology of the Genome-based (GP) and transcriptome-based (TP) probes on chiton transcriptomes. Branches that conflict with taxonomic expertise (and may indicate potential issues) are highlighted in red. Nodes without full local posterior probabilities (Local PP) or bootstrap values are labeled accordingly.


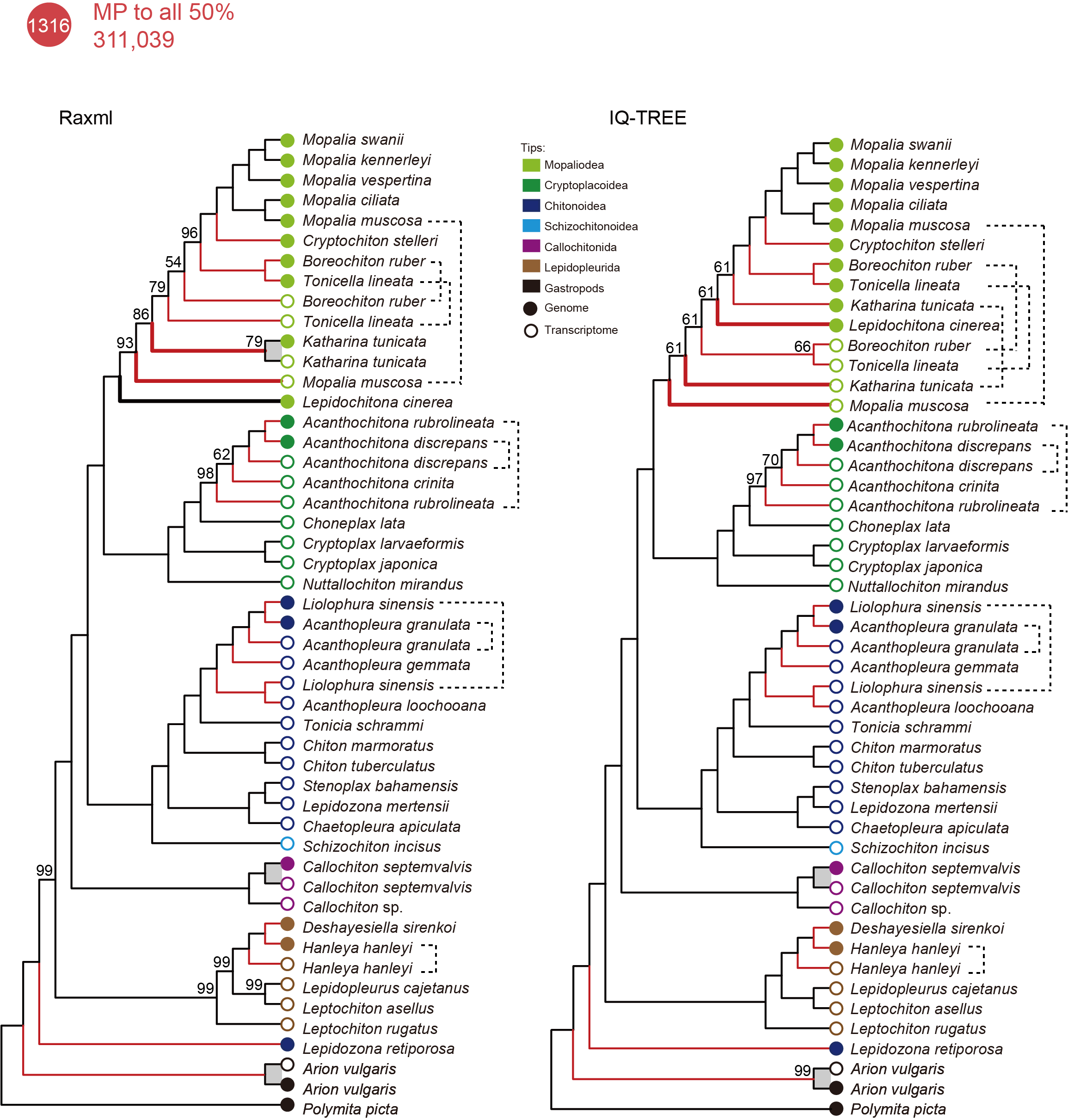


**Figure S10** Concatenated species tree for all chitons using chiton mixed probes (MP). Nodes without full bootstrap values are labeled accordingly. Branches that conflict with taxonomic expertise (and may indicate potential issues) are highlighted in red. Black dashed lines highlight species pairs that should be grouped together. The correct recovery of species-pairs connected by gray blocks.


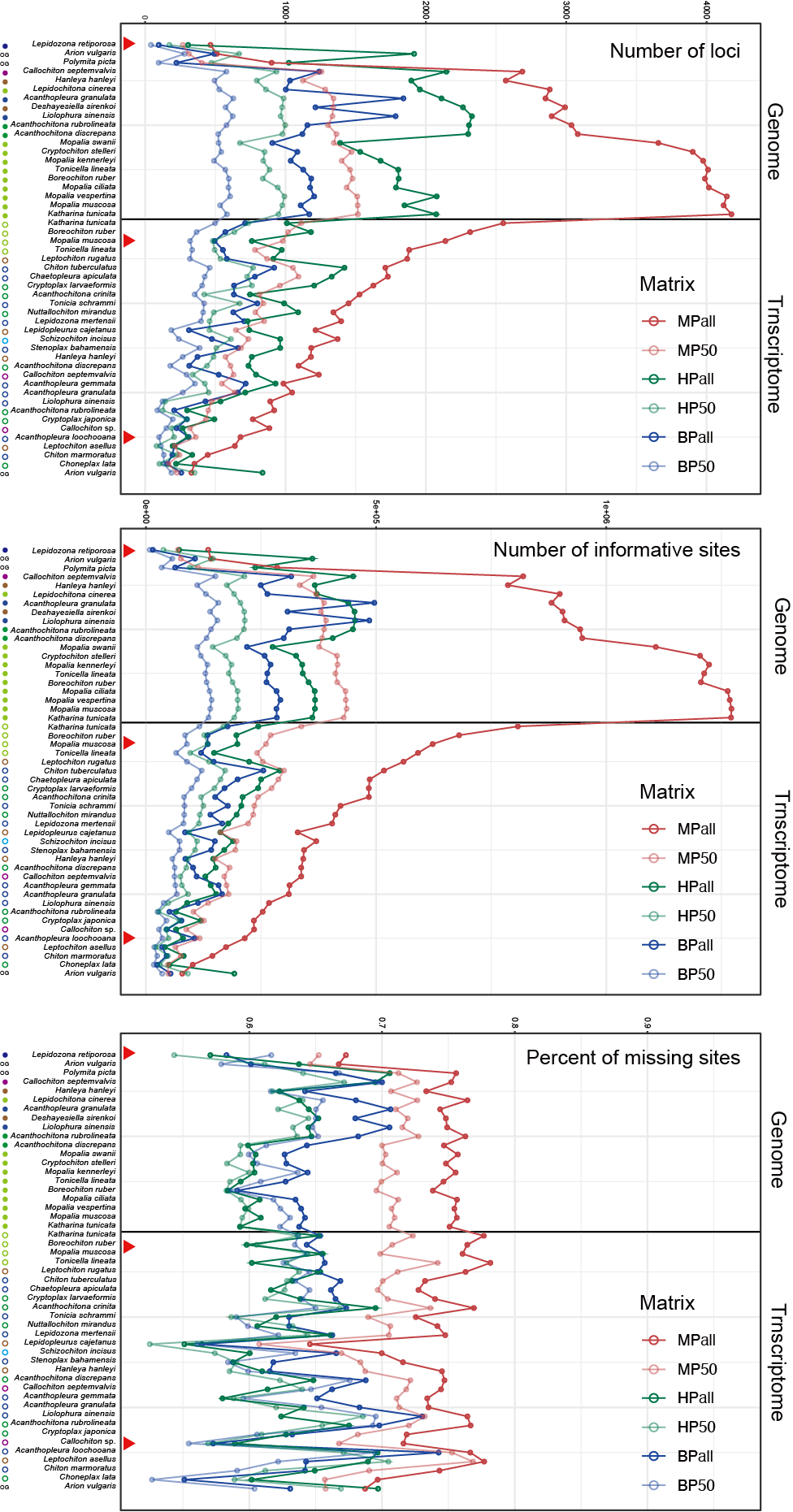


**Figure S11** Number of UCE loci, cumulative informative length, and proportion of informative sites (calculated as the proportion of non- “-” sites over the total sites) per species in phylogenetic matrices of different occupancy levels for MP (Mixed chiton), HP (Heterobranch gastropods), and BP (Bivalves) probe sets.


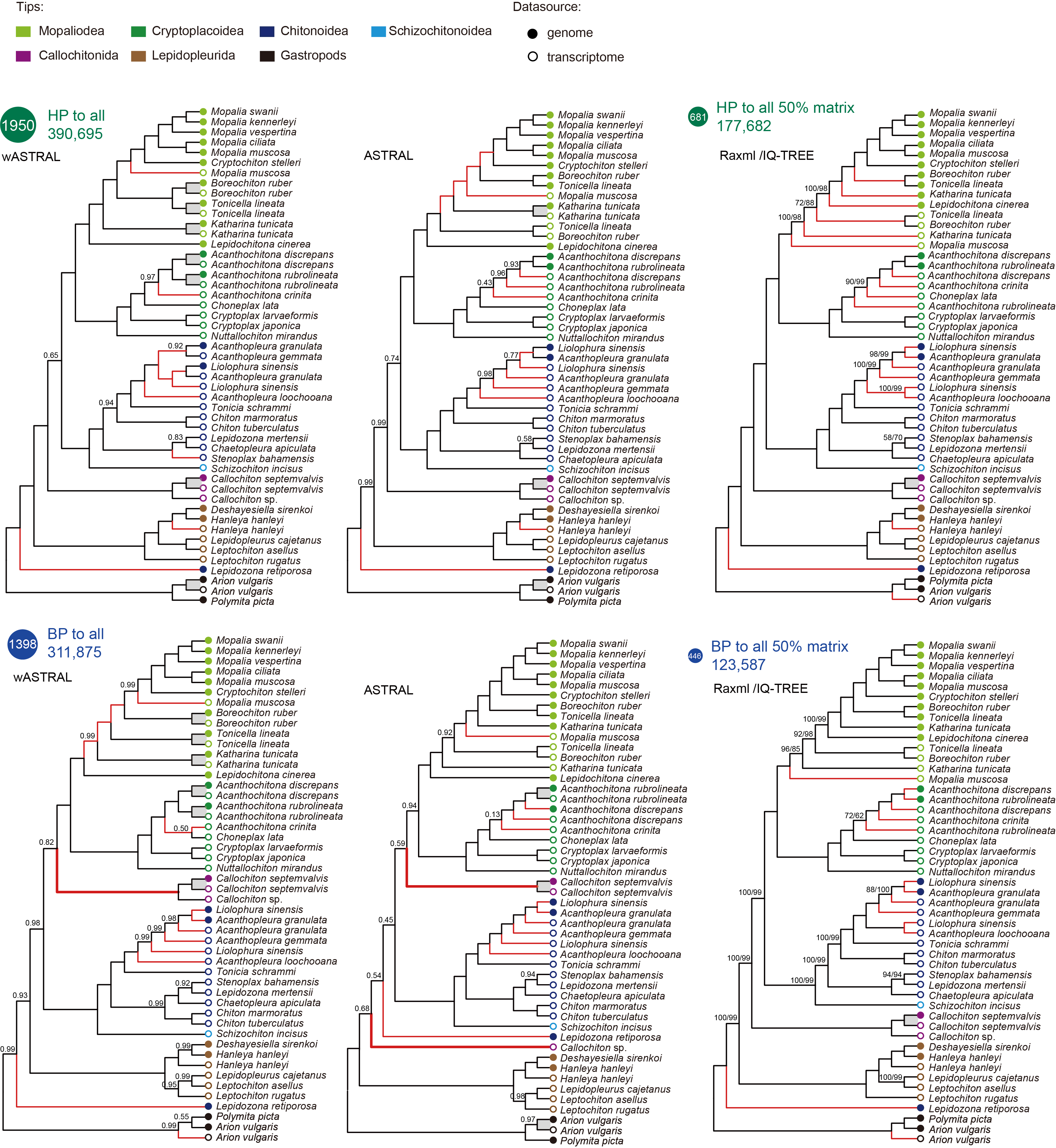


**Figure S12** Concatenated and coalescent species tree for all chitons using Gastropoda (Heterobranchia, HP) probes and Bivalvia probes (BP). Nodes without full local posterior probabilities (Local PP) or bootstrap values are labeled accordingly. Branches that conflict with taxonomic expertise (and may indicate potential issues) are highlighted in red. The correct recovery of species-pairs connected by gray blocks.


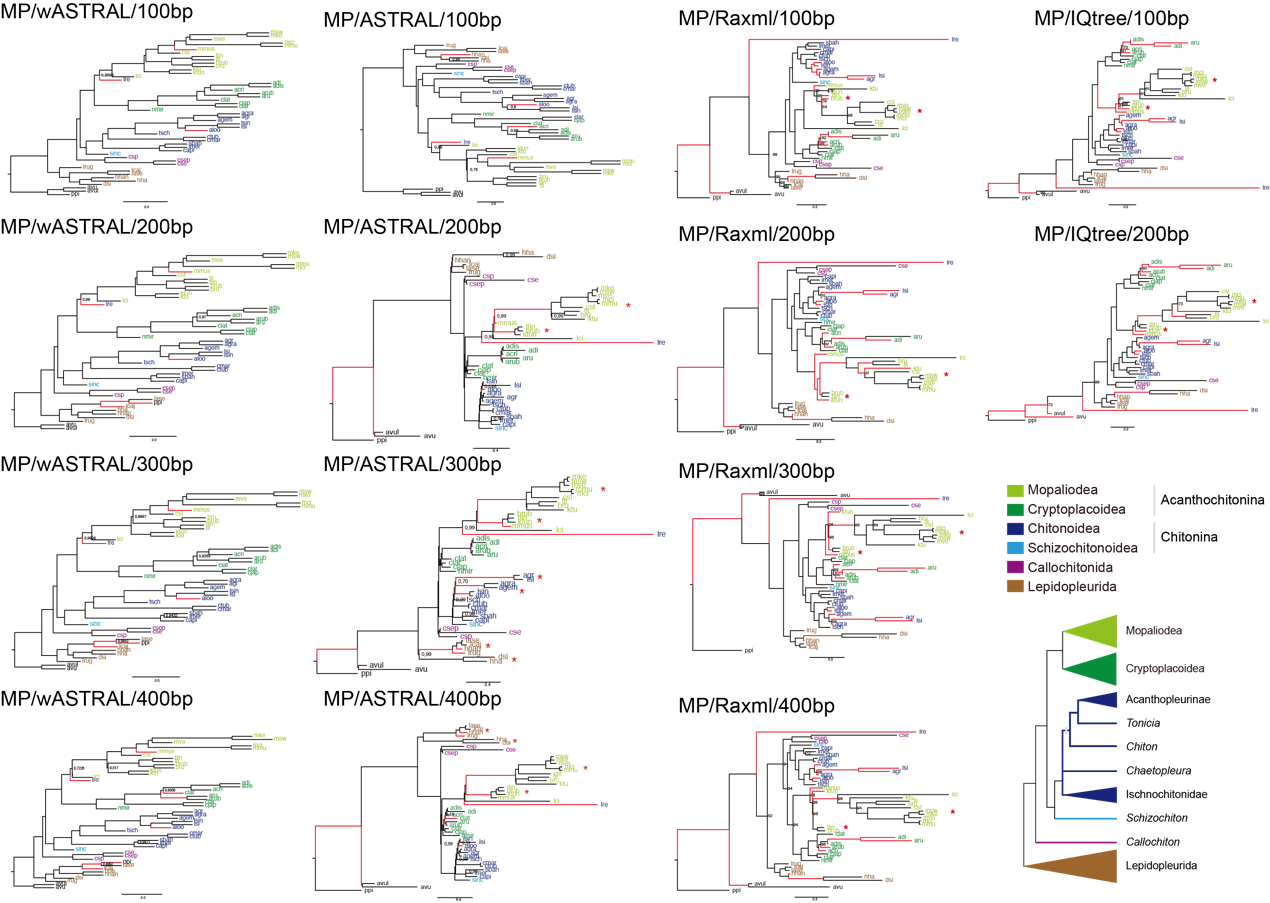


**Figure S13** Phylogenetic trees inferred using the mixed probes (MP) with different flanking sequence lengths. Species names are represented by the first letter of the genus followed by the first two letters of the species name for genome data, and by the first letter of the genus followed by the first three letters of the species name for transcriptome data. Nodes without full local posterior probabilities (Local PP) or bootstrap values are labeled accordingly. The IQTree analyses for flanking sequence lengths of 300 and 400 bp consumed excessive CPU time. We therefore terminated the runs, considering them to be of limited interpretive value. Red asterisks highlight cases where data of the same type cluster together. Branches that conflict with taxonomic expertise (and may indicate potential issues) are highlighted in red. For a summary of this figure, please refer to main text Figure 3.

**
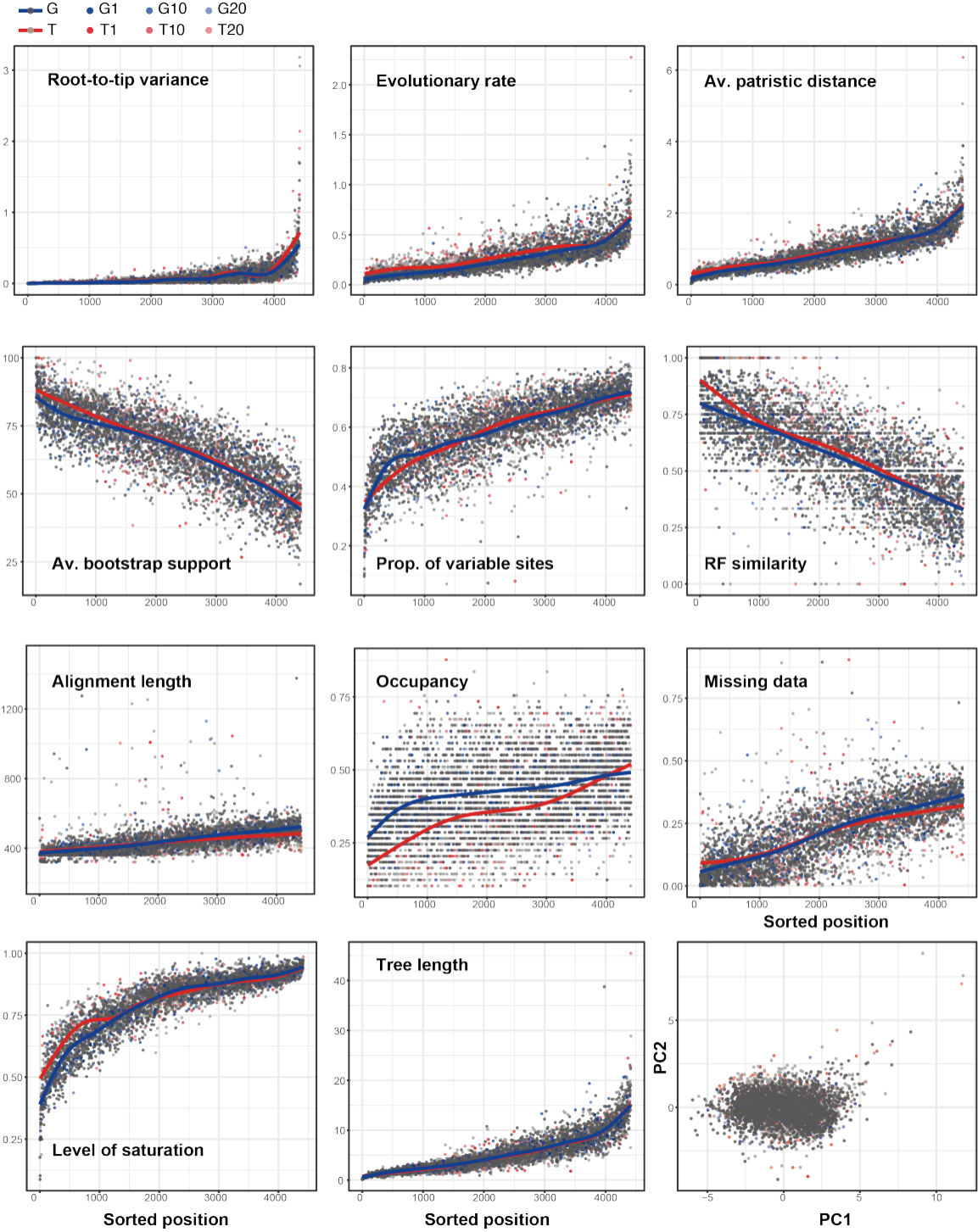
**

**Figure S14** Properties of phylogenomic loci captured by MP (Mixed chiton probes). Dark gray dots represent loci captured by GP (genome-based), while light gray dots indicate those from TP (transcriptome-based). The PCA analysis in the lower right corner was used to find an axis of phylogenetic usefulness along which proxies for signal increase while sources of bias decrease. Based on the assessment of phylogenetic utility, we filtered out the top 1%, 10%, and 20% of outliers. Outliers derived from GP are shown in blue with varying opacity levels, while those from TP are shown in red. Accordingly, the blue line and red line represent the LOESS regression of all sites captured from GP and TP, respectively.

**
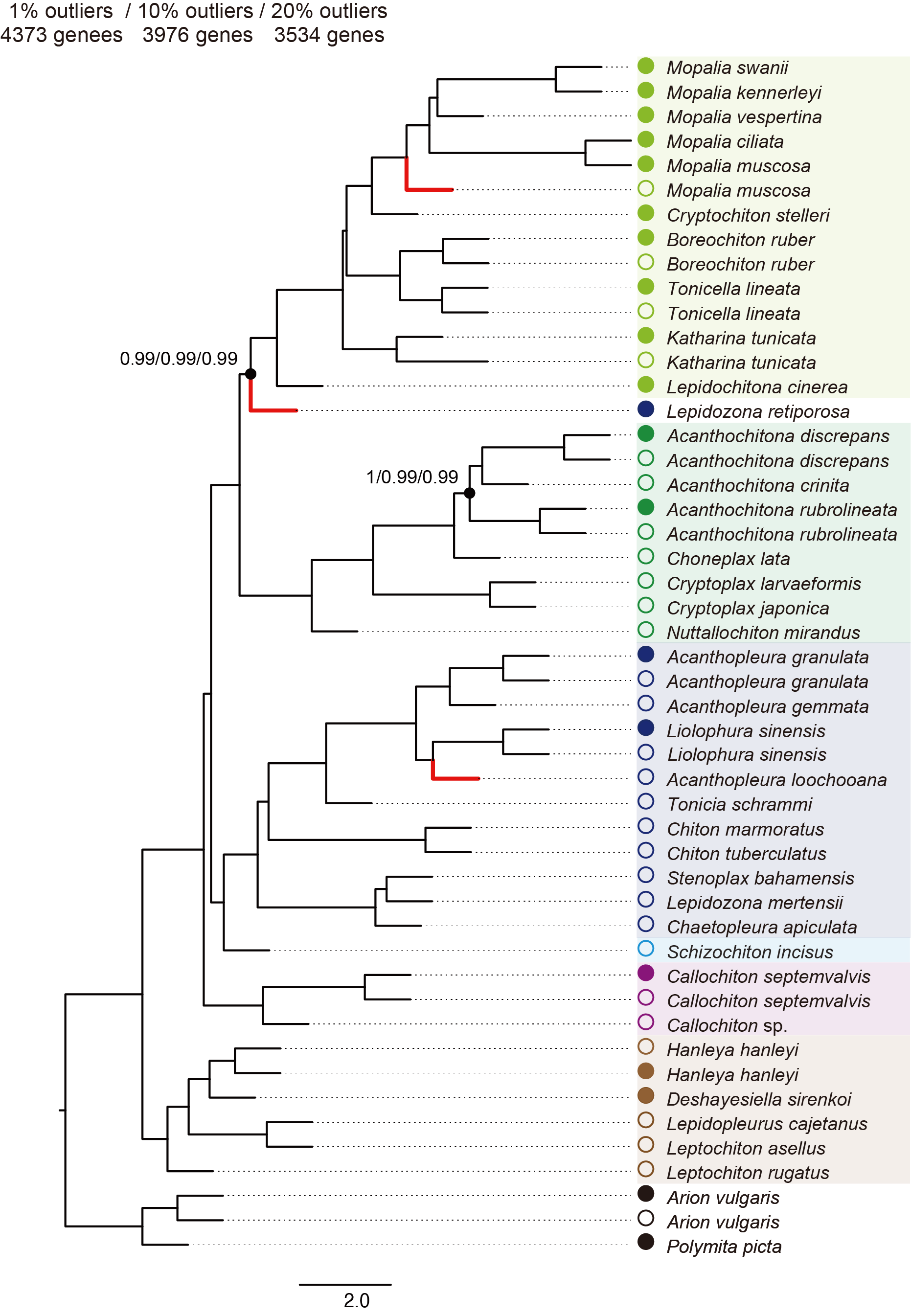
**

**FigS15** Performance of phylogeny under varying degrees of filtering. The tree is based on coalescent method using wASTRAL. Nodes with local posterior probabilities (Local PP) less than 1 are marked. Branches that conflict with taxonomic expertise (and may indicate potential issues) are highlighted in red.

**Supplementary Tables**

**Table S1 RNAseq reads used in transcriptome-based probe design and in silico analyses.**

| **Order** | **Family** | **Species** | **Location** | **Tissues** | **BioSample** | **Reads Accession Number** |
| --- | --- | --- | --- | --- | --- | --- |
| Callochitonida | Callochitonidae | *Callochiton septemvalvis* | Spain: Tossa de Mar | foot | SAMN16677970 | SRR13010089 |
| Callochitonida | Callochitonidae | *Callochiton sp.* | Antarctica: Ross Sea | whole | SAMN14765619 | SRR11674125 |
| Chitonida | Acanthochitonidae | *Acanthochitona crinita* | France: Coast Roscoff | whole | SAMN06141845 | SRR5110525 |
| Chitonida | Acanthochitonidae | *Acanthochitona rubrolineata* | China:Qingdao | larvae | SAMN10743606 | SRR8442642 |
| Chitonida | Acanthochitonidae | *Acanthochitona rubrolineata* | China:Qingdao | whole | SAMN10743605 | SRR8442643 |
| Chitonida | Chitonidae | *Acanthopleura gemmata* | Australia: Queensland, Heron Island | mantle | SAMN14765617 | SRR11674127 |
| Chitonida | Chitonidae | *Acanthopleura gemmata* | Australia: Queensland, Heron Island | girdle | SAMN14765616 | SRR11674128 |
| Chitonida | Chitonidae | *Acanthopleura gemmata* | Australia: Queensland, Heron Island | girdle, shell, secreting mantle | SAMN14765615 | SRR11674129 |
| Chitonida | Chitonidae | *Acanthopleura granulata* | USA:Florida, Florida Keys, near Harry Harris State Park | radula | SAMN14656768 | SRR11584250 |
| Chitonida | Chitonidae | *Acanthopleura granulata* | USA:Florida, Florida Keys, near Harry Harris State Park | radula | SAMN14656767 | SRR11584251 |
| Chitonida | Chitonidae | *Acanthopleura granulata* | USA:Florida, Florida Keys, near Harry Harris State Park | radula | SAMN14656766 | SRR11584252 |
| Chitonida | Chitonidae | *Acanthopleura granulata* | USA:Florida, Florida Keys, near Harry Harris State Park | radula | SAMN14656765 | SRR11584253 |
| Chitonida | Chitonidae | *Acanthopleura granulata* | USA:Florida, Florida Keys, near Harry Harris State Park | gonad | SAMN14656772 | SRR11584254 |
| Chitonida | Chitonidae | *Acanthopleura granulata* | USA:Florida, Florida Keys, near Harry Harris State Park | ctenidia | SAMN14656771 | SRR11584255 |
| Chitonida | Chitonidae | *Acanthopleura granulata* | USA:Florida, Florida Keys, near Harry Harris State Park | foot | SAMN14656770 | SRR11584256 |
| Chitonida | Chitonidae | *Acanthopleura granulata* | USA:Florida, Florida Keys, near Harry Harris State Park | girdle | SAMN14656769 | SRR11584257 |
| Chitonida | Chitonidae | *Acanthopleura loochooana* | China:Fujian | mantle, foot | SAMN21190202 | SRR15694065 |
| Chitonida | Chitonidae | *Acanthopleura loochooana* | China:Fujian | mantle, foot | SAMN21190201 | SRR15694066 |
| Chitonida | Chitonidae | *Acanthopleura loochooana* | China:Fujian | mantle, foot | SAMN21190200 | SRR15694067 |
| Chitonida | Chitonidae | *Acanthopleura loochooana* | China:Fujian | mantle, foot | SAMN21190199 | SRR15694068 |
| Chitonida | Chitonidae | *Acanthopleura loochooana* | China:Fujian | mantle, foot | SAMN21190198 | SRR15694069 |
| Chitonida | Chitonidae | *Acanthopleura loochooana* | China:Fujian | mantle, foot | SAMN21190197 | SRR15694070 |
| Chitonida | Chaetopleuridae | *Chaetopleura apiculata* | USA: Massachusetts, Woods Hole | girdle | SAMN14765620 | SRR11674124 |
| Chitonida | Chitonidae | *Chiton marmoratus* | British Virgin Islands: St. Thomas | decalcified valve | SAMN14765613 | SRR11674135 |
| Chitonida | Chitonidae | *Chiton tuberculatus* | British Virgin Islands: St. Thomas | radula | SAMN14765614 | SRR11674134 |
| Chitonida | Acanthochitonidae | *Choneplax lata* | Belize: Carrie Bow Bay | foot | SAMN16677971 | SRR13010088 |
| Chitonida | Cryptoplacidae | *Cryptoplax japonica* | Japan | foot | SAMN16677973 | SRR13010086 |
| Chitonida | Cryptoplacidae | *Cryptoplax larvaeformis* | Australia: Queensland, Heron Island | shell, secreting mantle | SAMN14765618 | SRR11674126 |
| Chitonida | Mopaliidae | *Katharina tunicata* | USA: Washington State, Friday Harbor, Cattle Point | radula | SAMN14765625 | SRR11674131 |
| Chitonida | Ischnochitonidae | *Lepidozona mertensii* | USA: Washington State, Friday Harbor, Dead Man's Bay | radula | SAMN14765626 | SRR11674130 |
| Chitonida | Chitonidae | *Liolophura sinensis* | Hong Kong:Kau Sai Chau | whole | SAMN35319769 | SRR24694061 |
| Chitonida | Chitonidae | *Liolophura sinensis* | Hong Kong:Kau Sai Chau | whole | SAMN35319768 | SRR24694062 |
| Chitonida | Chitonidae | *Liolophura sinensis* | Hong Kong:Kau Sai Chau | whole | SAMN35319767 | SRR24694063 |
| Chitonida | Chitonidae | *Liolophura sinensis* | Hong Kong:Kau Sai Chau | whole | SAMN35319766 | SRR24694064 |
| Chitonida | Chitonidae | *Liolophura sinensis* | Hong Kong:Kau Sai Chau | whole | SAMN35319765 | SRR24694065 |
| Chitonida | Mopaliidae | *Mopalia muscosa* | USA: Washington,Friday Harbor,Cattle Point | radula | SAMN14648874 | SRR11577121 |
| Chitonida | Mopaliidae | *Nuttallochiton mirandus* | Antarctica: Weddell Sea | girdle | SAMN14765623 | SRR11674133 |
| Chitonida | Ischnochitonidae | *Stenoplax bahamensis* | Belize: Carrie Bow Bay | foot | SAMN16677972 | SRR13010087 |
| Chitonida | Tonicellidae | *Tonicella lineata* | USA: Washington,Friday Harbor,Dead Man's Bay | radula | SAMN14649070 | SRR11577222 |
| Chitonida | Tonicellidae | *Tonicella lineata* | USA: Washington | mantle | SAMN08775006 | SRR6926331 |
| Chitonida | Chitonidae | *Tonicia schrammi* | USA: Florida, Panacea, Big Bend area | decalcified valves | SAMN14765624 | SRR11674132 |
| Chitonida | Acanthochitonidae | *Acanthochitona discrepans* | Northern Ireland | foot, viscera, radula, perinotum, and shell | SAMN41504957 | SRX27729828 |
| Chitonida | Acanthochitonidae | *Acanthochitona discrepans* | Northern Ireland | foot, viscera, radula, perinotum, and shell | SAMN41504957 | SRX24661709 |
| Chitonida | Schizochitonidae | *Schizochiton incisus* | China: South China Sea | girdle | SAMN32085629 | SRR22559962 |
| Lepidopleurida | Hanleyidae | *Hanleya hanleyi* | Norway: Bergen | mantle | SAMN14765621 | SRR11674123 |
| Lepidopleurida | Leptochitonidae | *Lepidopleurus cajetanus* | missing | muscle | SAMN10483041 | SRR8245857 |
| Lepidopleurida | Leptochitonidae | *Leptochiton asellus* | Northern Ireland: Queens University Marine Lab | mantle | SAMN14765622 | SRR11674122 |
| Lepidopleurida | Leptochitonidae | *Leptochiton asellus* | Northern Ireland: Ballyhenry Island | shell edge | SAMN32105719 | SRR22572831 |
| Lepidopleurida | Leptochitonidae | *Leptochiton asellus* | Northern Ireland: Ballyhenry Island | viscera | SAMN32105718 | SRR22572832 |
| Lepidopleurida | Leptochitonidae | *Leptochiton asellus* | Northern Ireland: Ballyhenry Island | aesthetes | SAMN32105717 | SRR22572833 |
| Lepidopleurida | Leptochitonidae | *Leptochiton asellus* | Northern Ireland: Ballyhenry Island | perinotum | SAMN32105716 | SRR22572834 |
| Lepidopleurida | Leptochitonidae | *Leptochiton asellus* | Northern Ireland: Ballyhenry Island | foot | SAMN32105715 | SRR22572835 |
| Lepidopleurida | Leptochitonidae | *Leptochiton rugatus* | USA: Reid Rock, Friday Harbor, WA | whole | SAMN03098848 | SRR1611558 |

**Table S2 Number of conserved loci shared among the reference genome *Acanthochitona discrepans* and chiton genomes.**

Criteria chosen in the design of the probe are bolded.

| **Loci shared by the reference and taxa** | **Orthologous loci (with a sequence divergence < 5%) shared among each genome** | **Temporary probe sets targeted loci** |
| --- | --- | --- |
| 1 | 1,216,243 | 4,726 |
| 2 | 463,344 | 4,385 |
| 3 | 223,197 | 4,224 |
| 4 | 169,263 | 4,140 |
| 5 | 135,113 | 4,067 |
| 6 | 110,349 | 4,010 |
| 7 | 90,967 | 3,948 |
| 8 | 75,268 | 3,901 |
| 9 | 61,518 | 3,850 |
| 10 | 49,606 | 3,796 |
| 11 | 39,186 | 3,743 |
| 12 | 30,015 | 3,685 |
| 13 | 22,060 | 3,606 |
| 14 | 15,437 | 3,490 |
| 15 | 9,972 | **3,333** |
| 16 | **5,756** | 2,997 |
| 17 | 2,788 | 2,278 |
| 18 | 936 | 990 |
| 19 | 186 | 279 |
| 20* (plus *Arion vulgaris* as outgroup) | - | 34 |

**Table S3 Conserved locus shared among the reference transcriptome *Acanthochitona rubrolineata* and chiton transcriptomes.**

Criteria chosen in the design of the probe are bolded.

| **Loci shared by the reference and taxa** | **Orthologous loci (with a sequence divergence < 5%) shared among each transcriptome** | **Temporary probe sets targeted loci** |
| --- | --- | --- |
| 1 | 1,000,157 | 8,063 |
| 2 | 492,394 | 7,813 |
| 3 | 347,082 | 7,577 |
| 4 | 260,477 | 7,331 |
| 5 | 208,369 | 7,086 |
| 6 | 172,603 | 6,835 |
| 7 | 144,981 | 6,543 |
| 8 | 122,583 | 6,224 |
| 9 | 103,597 | 5,870 |
| 10 | 87,238 | 5,453 |
| 11 | 72,842 | 4,975 |
| 12 | 60,272 | 4,406 |
| 13 | 49,419 | 3,803 |
| 14 | 40,098 | 3,165 |
| 15 | 32,093 | **2,524** |
| 16 | 25,404 | 1,961 |
| 17 | 19,743 | 1,429 |
| 18 | 15,106 | 922 |
| 19 | 11,454 | 551 |
| **20** | **8,460** | 303 |
| 21 | 6,042 | 145 |
| 22 | 4,127 | 63 |
| 23 | 2,645 | 25 |
| 24 | 1,646 | 8 |
| 25 | 964 | 4 |
| 26 | 476 | 0 |
| 27 | 175 | 0 |
| 28 | 36 | 0 |
| 29* (plus *Arion vulgaris* as outgroup) | - | 0 |

**Table S4 Alignment matrix statistics from different UCE data sets.**

GP: Genomic probe set; TP: Transcriptomic probe set; MP: Mixed probe set; GP: Gastropoda probe set; BP: Bivalvia probe set.

| **Data set** | **% matrix** | **#loci** | **Alignment length** | **Informative sites length** | **Mean species occupancy** |
| --- | --- | --- | --- | --- | --- |
| **Probes to Genomes (n=20)** |  |  |  |  |  |
| GP | 75% | 1,827 | 814,164 | 350,107 | 16.43 |
| GP | 50% | 2,852 | 1,239,869 | 498,032 | 15.01 |
| GP | all | 3,049 | 1,317,781 | 511,516 | 14.52 |
| TP | 50% | 906 | 399,774 | 157,376 | 13.57 |
| TP | all | 1,339 | 570,342 | 183,919 | 11.13 |
| **Probes to Transcriptomes (n=29)** |  |  |  |  |  |
| GP | 50% | 250 | 94,555 | 32,216 | 15.77 |
| GP | all | 2,302 | 846,389 | 179,200 | 8.3 |
| TP | 50% | 533 | 202,810 | 77,401 | 16.13 |
| TP | all | 1,542 | 583,011 | 178,359 | 10.92 |
| **Probes to all Genomes and transcriptomes (n=49)** |  |  |  |  |  |
| MP | 50% | 1,316 | 628,607 | 311,039 | 27.87 |
| MP | all | 4,584 | 2,027,027 | 855,400 | 19.02 |
| HP | 50% | 681 | 363,257 | 177,682 | 28.54 |
| HP | all | 1,950 | 963,292 | 390,695 | 18.65 |
| BP | 50% | 446 | 242,658 | 123,587 | 27.94 |
| BP | all | 1,398 | 708,072 | 311,875 | 18.47 |
